# Identification and study of new NF‐κB‐inducing kinase ligands derived from the imidazolone scaffold

**DOI:** 10.1002/ardp.202400614

**Published:** 2024-11-27

**Authors:** Francisco Maqueda‐Zelaya, Lara Valiño‐Rivas, Ana Milián, Sara Gutiérrez, José Luis Aceña, Javier Garcia‐Marin, Mª Dolores Sánchez‐Niño, Juan J. Vaquero, Alberto Ortiz

**Affiliations:** ^1^ Departamento de Química Orgánica y Química Inorgánica, Instituto de Investigación Química “Andrés M. Del Río” (IQAR) Universidad de Alcalá (IRYCIS) Alcalá de Henares, Madrid Spain; ^2^ Departamento de Nefrología e Hipertensión IIS‐Fundación Jiménez Díaz UAM Madrid Spain; ^3^ RICORS2040 Madrid Spain; ^4^ Departamento de Farmacología, Facultad de Medicina Universidad Autónoma de Madrid Madrid Spain

**Keywords:** imidazolone, MAP3K14, NF‐κB‐inducing kinase, NIK, noncanonical pathway

## Abstract

Chronic kidney disease (CKD) is a growing health concern, projected to be a major cause of death by 2040, due to an increasing risk of acute kidney injury (AKI). Systems biology‐derived data suggest that the unmet need for an orally available drug to treat AKI and improve CKD outcomes may be addressed by targeting kidney inflammation and, specifically, nuclear factor κB‐inducing kinase (NIK), a key signaling molecule that activates the noncanonical nuclear factor κB (NF‐κB) pathway. We have prepared and identified a small family of imidazolone derivatives that bind NIK and inhibit the noncanonical NF‐κB activation pathway. The introduction of heterocyclic substituents in position 2 of the imidazolone core provides compounds with affinity against human NIK. Three candidates, with best affinity profile, were tested in phenotypic experiments of noncanonical NF‐κB activation, confirming that the derivative bearing the 4‐pyridyl ring can inhibit the processing of NFκB p100 to NFkB2 p52, which is NIK‐dependent in cultured kidney tubular cells. Finally, exhaustive docking calculations combined with molecular dynamics studies led us to propose a theoretical binding mode and rationalize affinity measures, in which the aminopyridine motif is a key anchoring point to the hinge region thanks to several hydrogen bonds and the interaction of heterocyclic rings in position 2 with Ser476 and Lys482. Our result will pave the way for the development of potential drug candidates targeting NIK in the context of CKD.

## Graphical abstract

A new family of NF‐κB‐inducing kinase (NIK) ligands bearing an imidazolone moiety were synthesized. The best compound showed a dose‐dependent inhibition of p100 to p50 processing in cellular assays, a key step in nuclear factor κB (NF‐κB) activation. Based on computational techniques, a plausible binding mode is proposed.

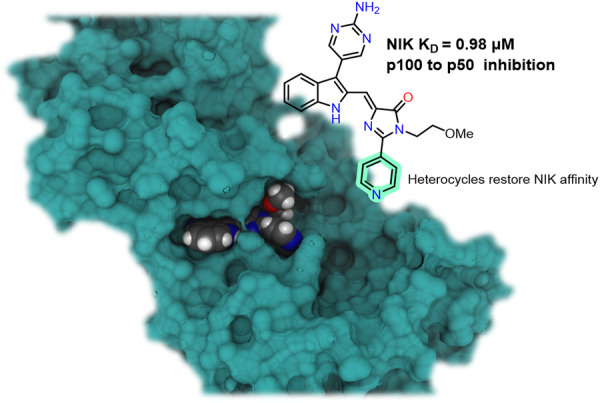

## 1 INTRODUCTION

Chronic kidney disease (CKD) is one of the fastest‐growing causes of death and is projected to be the fifth global cause of premature death by 2040.^[^ ^1^ ^]^ CKD can be defined as abnormalities of kidney structure or function, which have an impact on health. It is diagnosed when the glomerular filtration is <60 mL/min/1.73 m^2^ or when there is evidence of renal damage.^[^ ^2^ ^]^ Patients with CKD are prone to suffer acute kidney injury (AKI), which additionally contributes to CKD progression and has a mortality that may approach 50.^[^ ^3^ ^]^ Up to date, there are no pharmacological tools to treat AKI or the AKI to CKD transition, and the residual risk for current therapy for CKD is still high. In consequence, further research is needed to understand this complex disease and pave the way for life‐saving treatments that restore kidney function or improve AKI or CKD outcomes.

CKD is commonly caused by diabetes, followed by vascular alterations such as glomerulonephritis and hypertension.^[^ ^1^ ^]^ However, independently of the cause, CKD progresses through a well‐defined series of pathophysiological events including irreversible nephron loss, microvascular damage, oxidative stress, and inflammation, ultimately leading to renal fibrosis.^[^ ^4^ ^]^ In fact, growing evidence indicates that renal inflammation plays a pivotal role in the origin and progression of renal fibrosis.^[^ ^3^, ^4^, ^5^ ^]^ Additionally, inflammation is a key contributor to AKI. Among the main players in the inflammatory process, the NF‐κB (nuclear factor kappa‐light‐chain‐enhancer of activated B cells) transcription factor controls the expression of a large number of proinflammatory genes and decreases the expression of tissue‐protective molecules.^[^ ^5^ ^]^ Mammalian NF‐κB consists of a family of structurally related proteins, including RelA (p65), c‐Rel, p50 (NF‐κB1), and p52 (NF‐κB2). These proteins share a common Rel homology domain, allowing them to interact and form homo‐ and heterodimers. Through this domain, NF‐κB transcription factors bind to κB sequence elements of mammalian genes related to inflammation.^[^ ^6^ ^]^ Under physiological conditions, NF‐κB dimers are normally sequestered in the cytoplasm by a family of inhibitors (IκBs) that forms inactive complexes, including p105 and p100, the precursor proteins of p50 and p52, respectively.^[^ ^6^ ^]^ Upon activation, proteasome‐mediated processing of these proteins generates mature and functional NF‐κB1 and NF‐κB2 transcription factors.^[^ ^7^ ^]^

NF‐κB activation can take place through two major and well‐defined pathways, canonical and noncanonical, related with the inducible processing p105 and p100, respectively. While classical NF‐κB activation occurs as a rapid and transient response to a wide range of stimuli that produce p105 cleavage, the noncanonical pathway implicates the slow processing of p100/RelB heterodimers to p52/RelB with the consequent prolonged activation of NF‐κB target genes.^[^ ^8^ ^]^ Interestingly, this noncanonical pathway has been related with AKI and the progression of CKD and renal fibrosis^[^ ^9^ ^]^ or renal injury^[^ ^10^ ^]^ among others.^[^ ^5^, ^8^, ^11^ ^]^ In this regard, the pharmacomodulation of the NF‐κB noncanonical pathway could represent a strategy for the treatment of these kidney‐related diseases.

**Figure 1:**
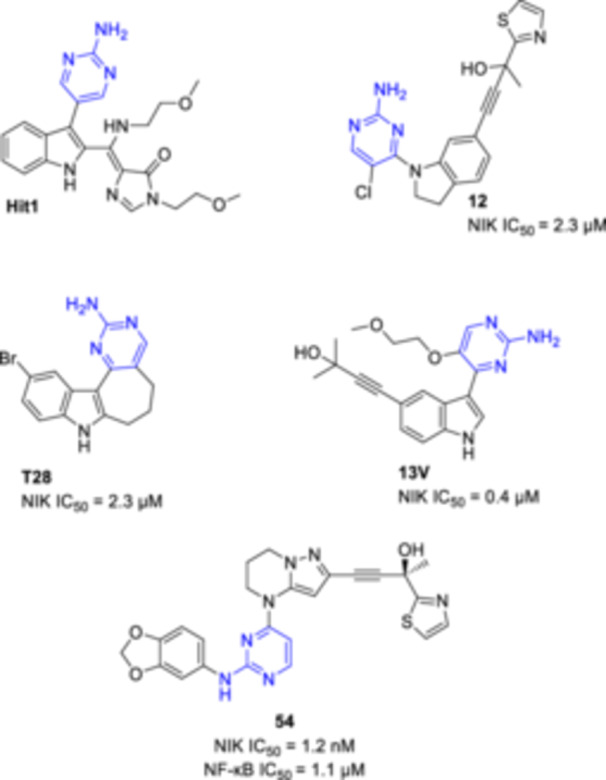
Inhibitors of nuclear factor κB (NF‐κB) signaling.

To search for new inhibitors of NF‐κB signaling, our research group screened a few examples of our in‐house library of heterocyclic compounds. This let us identify Hit1, an azolo‐imidazolidinone derivative that was able to inhibit the proteolytic cleavage of p100/RelB to p52/RelB in cultured murine kidney proximal tubular cells, a key cell target in AKI and CKD. Interestingly, this molecule bears an aminopyrimidine motif, a common fragment found in other active site‐directed kinase inhibitors, including the nuclear factor κB‐inducing kinase (NIK) (Figure 1).^[^ ^12^, ^13^, ^14^, ^15^, ^16^, ^17^ ^]^ This protein (also known as MAP3K14) is a serine/threonine kinase of 947 amino acids (in the human sequence) that triggers the activation of the NF‐κB noncanonical pathway.^[^ ^18^ ^]^ Due to its implication in this signaling axis, NIK has been proposed as a potential therapeutic target for kidney‐associated diseases including AKI and CKD.^[^ ^8^, ^19^ ^]^ The lack of a validated and clinically approved drug against NIK still represents an opportunity for drug discovery groups interested in the modulation of this protein in the context of CKD.

Considering these discoveries, we aimed to carry out a preliminary exploration of the imidazolone scaffold as potential inhibitor of the noncanonical NF‐κB signaling. We pursued novel and patentable promising anti‐inflammatory molecules with appropriate physicochemical parameters and drug‐like properties.

## 2 RESULTS AND DISCUSSION

### 2.1 Chemistry

**Scheme 1:**
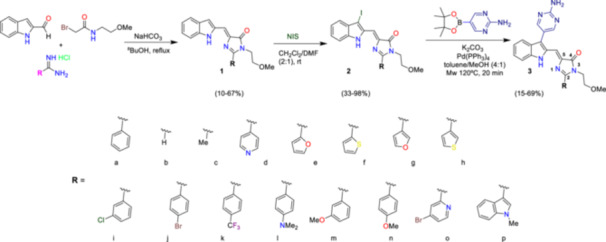
Synthesis of imidazolone derivatives.

To achieve hit expansion of the imidazolone Hit1 in the patentable chemical space, we directed our efforts toward introducing chemical diversity at position 2 of the imidazolone ring. We employed a multicomponent reaction previously set up by our lab for this purpose.^[^ ^20^ ^]^ Briefly, a mixture of indole‐2‐carboxaldehyde, an amide generated by condensation of bromoacetyl bromide with 2‐methoxyethylamine, and an appropriately substituted formamidine were refluxed until obtention of imidazolones **1** in low to moderate yields (Scheme 1). Diversification of position 2 of the imidazolone ring was achieved by preparing the corresponding formamidines or by purchasing those available from commercial sources. To attach the 4‐aminopyrimidine motif to compound **1**, we employed a Suzuki coupling. To this aim, previous functionalization was needed, so an iodine atom was easily introduced thanks to the treatment of **1** with NIS, yielding the corresponding halogenated derivatives **2** in very good yields. Finally, Suzuki coupling was carried out using different pinacol boronates under microwave irradiation using standard conditions to afford final products in moderate to good yields (Table 1).

**Table 1:** Final products and yields for the last reaction step.

Entry	Name	R substituent	Yield (%)	Purity (%)^a^
1	**3a**	phenyl	38	100
2	**3b**	H	23	98
3	**3c**	methyl	74	95
4	**3d**	4‐pyridyl	22	93
5	**3e**	2‐furyl	18	95
6	**3f**	2‐thienyl	59	99
7	**3g**	3‐furyl	30	97
8	**3h**	3‐thienyl	68	98
9	**3i**	3‐chlorophenyl	23	95
10	**3j**	4‐bromophenyl	49	93
11	**3k**	4‐(trifluoromethyl)phenyl	24	99
12	**3l**	4‐(dimethylamino)phenyl	69	97
13	**3m**	3‐methoxyphenyl	25	99
14	**3n**	4‐methoxyphenyl	15	91
15	**3o**	3‐bromo‐2‐pyridyl	22	72
16	**3p**	*N*‐methyl‐2‐indolyl	47	ND

Abbreviation: ND, not determined.

^a^ Purity measured by HPLC‐UV at 254 nm.

### 2.2 Pharmacology

#### 2.2.1 Binding experiments

As previously stated, the 4‐aminopyridine moiety is a common pattern found in NIK inhibitors, a pivotal enzyme involved in the noncanonical NF‐κB activation. Bearing this in mind, we wondered if our compounds were targeting human NIK, so we performed enzyme inhibition experiments using a commercial NIK inhibition kit (ADP‐Glo assay from Promega, see Supporting Information S2: Figure S1). Unfortunately, it was not possible to obtain reliable results, especially at high concentrations due to solubility constraints and nonlinear effects using the manufacturer's experimental conditions. It is worth noting that despite the potential PAINs alert from the Michael acceptor group, incubation of these compounds with glutathione and further analysis by HPLC and ^1^hydrogen nuclear magnetic resonance (H‐NMR) did not show the presence of any Michael adduct.^[^ ^21^ ^]^

Considering this, we decided to measure the affinity of our compounds against recombinant NIK using a competition binding assay provided by the company Eurofins. To our delight, we discovered that some imidazolone derivatives were active, showing interesting affinities in the low micromolar range and thus confirming our initial hypothesis (Table 2).

**Table 2:** Affinity measurement and drug‐like parameters of synthesized compounds.

Entry	Name	Apparent *K*_d_ (µm)	MW (g/mol)^a^	cLogP (RDKit)^a^	HBDonors^a^	HB receptors^a^	Lipinski^a^
1	**3a**	>10	438.49	3.48	6	2	Yes
2	**3b**	1.5	362.39	2.06	6	2	Yes
3	**3c**	5.1	376.42	2.45	6	2	Yes
4	**3d**	0.98	439.47	2.87	7	2	Yes
5	**3e**	2.8	428.45	3.07	7	2	Yes
6	**3f**	6.1	444.52	3.54	7	2	Yes
7	**3g**	4.5	428.45	3.07	7	2	Yes
8	**3h**	5.5	444.52	3.54	7	2	Yes
9	**3i**	>10	472.93	4.13	6	2	Yes
10	**3j**	>10	517.38	4.24	6	2	No
11	**3k**	>10	506.48	4.5	6	2	No
12	**3l**	>10	481.56	3.54	7	2	Yes
13	**3m**	>10	468.51	3.49	7	2	Yes
14	**3n**	>10	468.51	3.49	7	2	Yes
15	**3o**	ND	518.37	3.64	7	2	No
16	**3p**	ND	439.47	2.87	7	2	Yes

Abbreviation: ND, not determined.

^a^ Parameters calculated with RDKit software.

The introduction of aromatic or heteroaromatic rings in position 2 of the imidazolone core induces significant changes in apparent *K*_d_, which are dependent on substituent shape and volume as can be deduced from Table 2. Remarkably, substituted aromatic rings (entries 9–14) turned out to be inactive, which suggests that the space filled by these rings in the protein must be limited. Interestingly, the exchange of benzene ring (**3a**) with a classical isostere such as thiophen (**3f**) restores ligand binding. This tendency is also consistent with other bioisosteric replacements including furan and pyridine rings. This phenomenon seems to be related to the presence of a heteroatom in the aromatic ring that could establish extra interactions. Moreover, deletion or methyl substitution at this position is also tolerated but with a decrement in the affinity against the protein.

These preliminary SAR observations highlight the importance of this position in tuning the compound's affinity toward human NIK. Regarding drug‐like properties, all compounds displayed a good profile in terms of Lipinski parameters (see Table 2), being **3j**, **3k**, and **3o** the only outliers due to their molecular weight, exceeding 500 g/mol. Calculated LogP (RDKit) are also under 5, and values are compatible with oral absorption, thus reinforcing the interest of our compounds as future leads or potential drug‐like candidates.

#### 2.2.2 Cell viability

**Figure 2:**
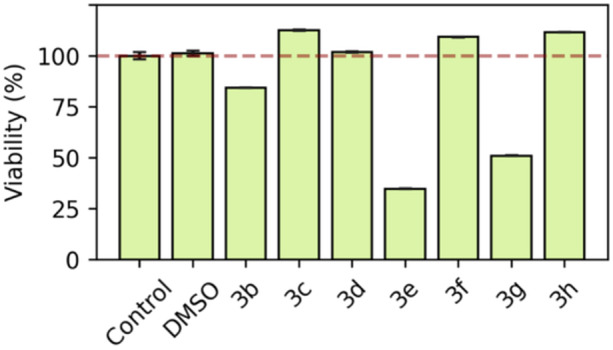
Cell viability assay (MTT) in HFF1 cell line at 10 µM final concentration for selected compounds. Experiments were performed in replicate (*n* = 4). Bars denote the mean percentage ± SD. HFF1, human foreskin fibroblast 1.

To conduct a preliminary safety assessment, we screened for potential cytotoxic effects with the MTT assay at a fixed concentration of 10 µM against a fibroblast healthy cell line (Figure 2). Based on binding results, we chose those compounds that exerted affinity against NIK, discovering that derivatives **3e** and **3g** were remarkably cytotoxic after 24 h. Nonetheless, **3b** and **3d**, which exhibited the best apparent Kd, did not show any toxicity under these conditions. Consequently, we concluded that most of our compounds were interesting for further consideration.

#### 2.2.3 Suppression of NIK/NF‐kB2 signaling

**Figure 3:**
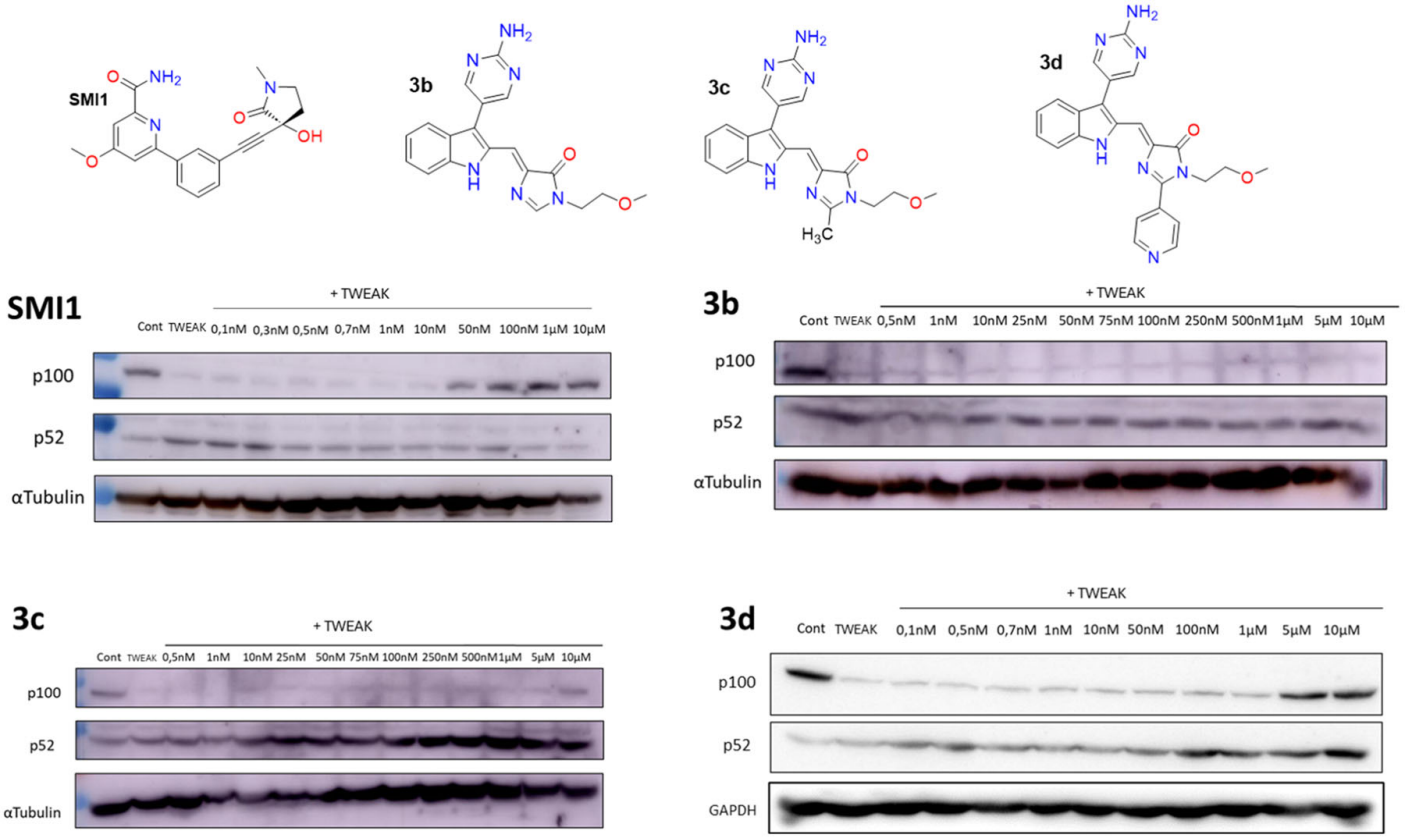
Assessment of the inhibitory effect of diverse compounds on NFkB2 activation in live cultured cells. For each compound, the structure and a representative Western blot are shown. Western blot of NF‐kB2 p100 (unprocessed form) and p52 (processed, active form) in MCT cells stimulated with TWEAK (100 ng/mL for 3 h), a cytokine known to activate the noncanonical nuclear factor κB (NFkB) pathway through NF‐κB‐inducing kinase (NIK)‐dependent p100 to p52 processing. Note that upon TWEAK stimulation NFkB2 p100 disappears and the NFkB2 p52 band increases, leading to a decrease in the NFkB2 p100/p52 ratio. MCT, medium‐chain triglycerides; NF‐kB2, Nuclear Factor kappa B subunit 2; TWEAK, tumor necrosis factor‐like weak inducer of apoptosis.

Once the safety of the best candidates was confirmed, we moved to the evaluation of the phenotypic effects in cellular models. As previously stated, NFkB2 p100 to p52 processing is a NIK‐dependent hallmark step in the noncanonical activation pathway of NF‐κB. So, we decided to examine whether our compounds could act as NIK inhibitors in a cellular environment and halt the production of p52. We chose, among the active binders, compounds **3b**, **3c**, and **3d** to explore their behavior in a phenotypic assay of inflammation responsiveness using a kidney tubular mast cell line (Figure 3). Furthermore, we included a commercial NIK inhibitor (SMI1) as a positive control for these experiments in the presence of tumor necrosis factor‐like weak inducer of apoptosis (TWEAK). This proinflammatory cytokine, a therapeutic target in kidney disease,^[^ ^22^ ^]^ is an endogenous activator of the noncanonical NF‐κB signaling pathway that promotes Nuclear Factor kappa B subunit 2 (NF‐κB2) p100 processing to p52 (Figure 3).^[^ ^19^ ^]^

As expected, SMI1 dose‐dependently preserved p100 levels, and the level of p52 dose‐dependently decreased, indicating the blockade of p100 proteolysis caused by inhibition of the cellular NIK activity and thus validating our experimental setup (Figure 3). Strikingly, compound **3b** turned out to be inactive under the same conditions, as p100 was fully processed into p52 at any concentration, indicating the absence of suppression of NIK/NF‐kB2 signaling. Regarding the methyl derivative **3c**, p100 preservation was clearly observed at 10 µM, which can be due to its low potency in cellular assays. However, **3d**, featuring a 4‐pyridyl fragment, exhibited a dose‐dependent p100 preservation, thus reinforcing the interest of this candidate as a valuable NIK inhibitor. This behavior can be related with the permeability and lipophilicity of the compounds (Table 2) since the best results in Western blot experiments were obtained for **3d**, which presented the highest cLogP (2.87), followed by **3c** (2.45) and **3b** (2.06).

### 2.3 Biomolecular modeling

**Figure 4:**
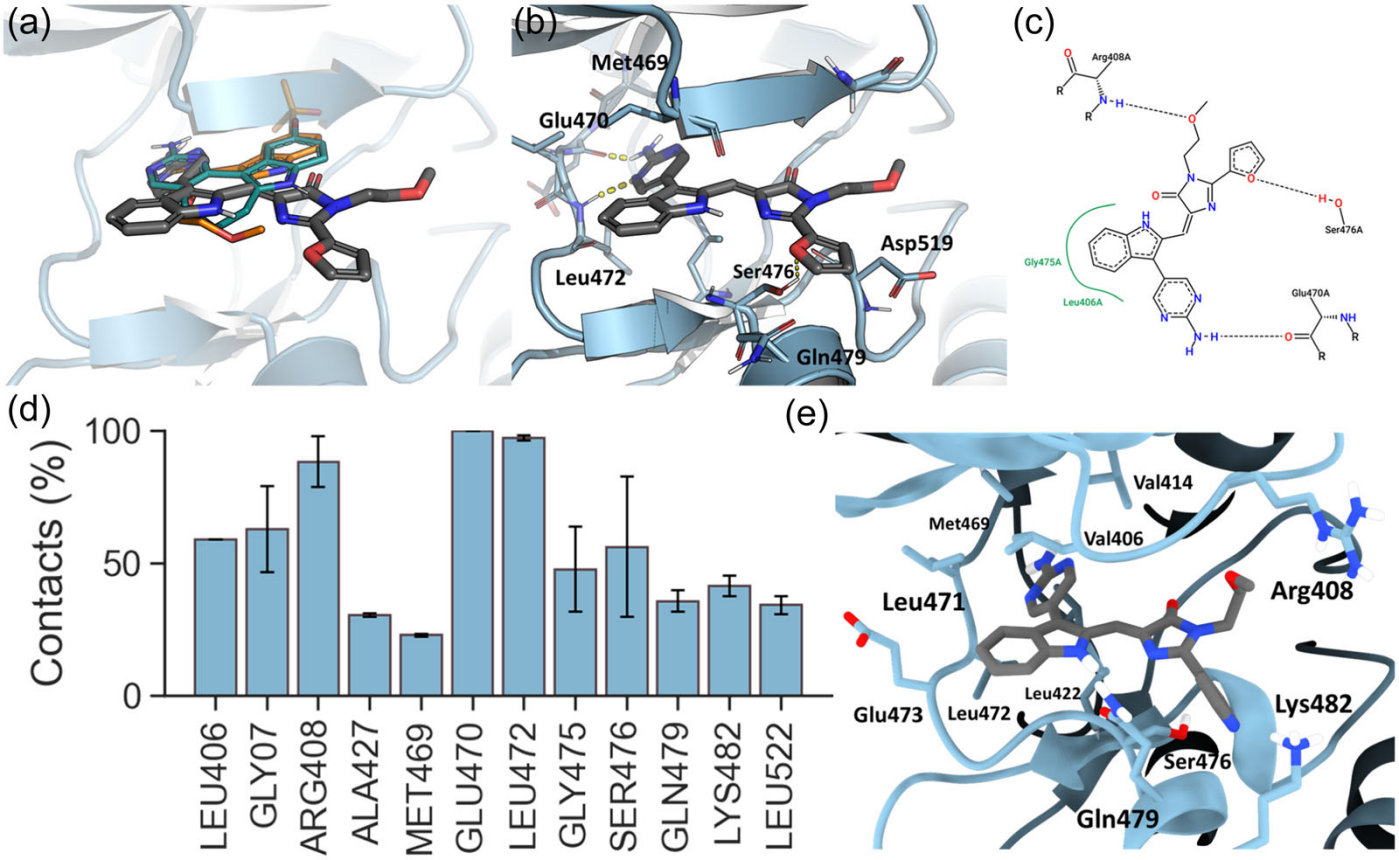
(a) Superimposition of best docking pose from **3e** (gray sticks) and NF‐κB‐inducing kinase (NIK inhibitors) **T28** (turquoise) and **13V** (orange) from Protein Data Base (PDBs): 4IDT and 4IDV, respectively.^[^ ^23^ ^]^ (b) Zoom‐view of docked **3e** inside NIK ATP pocket (hydrogen bonds are represented as yellow dashed lines). (c) PoserView diagram generated from docking results. (d) Percentage of contact (distance ≤4 Å) between ligand and protein residue atoms averaged between **3b** and **3d** molecular dynamics (MD) simulations. (e) Representative snapshot from MD simulation of **3d** and NIK.

Finally, to gain further insights into the binding mode between imidazolone derivatives and NIK, we next performed docking calculations using a homology model of human NIK in DFG‐in conformation. All synthesized ligands were easily docked inside the enzyme catalytic throat depicting two different binding modes that correlated with its classification as binders or not binders (see Supporting Information S2: Figure S2). Compounds with bulkier groups at position 2 of the imidazolone ring oriented their substituent toward the activation segment of the protein, while active binders placed the same group toward Ser476 and Gln479 residues at the entry of the ATP pocket. These observations suggest that big substituents in 2 hamper ligand binding to NIK since those compounds cannot reproduce the same binding mode as the binders. Therefore, our docking protocol was able to discriminate between binders or not binders in our congeneric family of compounds. Active compounds share a similar pattern in the binding mode as depicted in Figure 4, which is common and consistent with other inhibitors. The aminopyridine moiety is accommodated in the same fashion as previously described inhibitors (Figure 4a), establishing hydrogen bonds with the backbone of Glu470 and Leu472 from the hinge region and acting as a strong anchoring fragment (Figure 4b,c).^[^ ^23^ ^]^

Additional interactions are provided by hydrophobic contacts with those residues present in the adenine pocket including Gly475, Leu406, Ala427, Leu471, and Leu522. Regarding the 3‐methoxyamino lateral chain, it can establish a hydrogen bond with Arg408 –NH group from the backbone. Small aromatic substituents in position 2 of the imidazolone establish CH–π interactions with Ser476 or Asp519 sidechains in the solvent‐exposed region. For those derivatives with a heteroaryl ring such as furan, pyridine, or thiophene (compounds **3d** to **3g**), an extra hydrogen bond can be found between Ser476 –OH group and the heteroatom, which accounts for their affinity compared with methyl and hydrogen derivatives (**3b** and **3d**, respectively).

To inform about the complex stability and dynamical behavior of protein–ligand complexes, we selected **3b** and **3d** derivatives for molecular dynamics (MD) studies because of their high affinity toward NIK. Classical MD simulation can provide a realistic picture of protein–ligand interactions and their evolution, and they are also common tools to refine docking poses. Both derivatives were simulated for 250 ns to assess the feasibility of the binding mode using experimental‐like conditions (explicit water, 298 K and KCl ions).

Complexes were stable along the simulation time, according to time‐evolution RMSD profile from protein backbone atoms (see Supporting Information S2: Figure S3). Additionally, both ligands remained firmly bound to the protein with mean RMSD value of 2.26 ± 0.73 and 2.05 ± 0.49 Å for **3b** and **3d**, respectively, across the trajectory.

Regarding ligand stability and interactions, we observed a good percentage of contacts with all surrounding residues in the ATP pocket, thus supporting the stability of docking poses and confirming previously described interactions with the protein. Especially relevant are hydrophobic contacts with lateral chains of residues Glu470, Leu472, Arg408, Leu406, and Leu522 (Figure 4d). Some fluctuations were observed in the lateral methoxy‐amino‐ethyl chain which explores the outer space of the protein because of its flexibility to briefly interact with a hydrogen bond between the –NH_2_^+^ from Arg408 guanidinium group and the –OCH_3_. Overall, the number of hydrogen bonds formed between protein and ligands fluctuated during trajectories but always maintaining at least the two bonds associated with the aminopyridine fragment, which significantly contributed to maintaining the theoretical binding mode (Figure 4e and Supporting Information S2: Figure S3). It is noteworthy that, in the case of **3d**, a stable hydrogen bond between the nitrogen atom of the pyridine ring and NH_3_^+^ from Lys482 side chain was observed during the simulation, which accounts for its higher affinity.

Finally, we investigated the theoretical affinity for our models using WaterSwap absolute binding free energy calculations. This methodology employs a reaction coordinate that swaps a ligand bound to a protein with an equivalent volume of bulk water with the key advantage of using a single simulation which also incorporates protein flexibility through Monte Carlo simulations.^[^ ^24^, ^25^ ^]^ Our calculations yielded a mean predicted binding energy of –15.32 ± 0.78 and –17.95 ± 0.72 kcal/mol for **3b** and **3d**, respectively (see Supporting Information S2: Figure S4). So, we confirmed by estimated binding free energies that both compounds can act as NIK binders in the predicted binding mode. Therefore, WaterSwap simulations successfully differentiated between the two ligands in terms of affinity, thereby validating our whole biomolecular modeling approach.

## 3 CONCLUSIONS

AKI and CKD represent health challenges for the incoming years due to their high expected prevalence, especially in high‐income countries, where it is a significant problem for the elderly. Therefore, the development of validated and effective pharmacological treatments remains a critical area of research.

Targeting inflammation during CKD early stages can ameliorate its progression and kidney degeneration such as fibrosis, thus becoming a promising therapeutic strategy, especially through the modulation of the noncanonical activation pathway. The imidazolone derivatives have the potential to interact with molecular targets of NF‐κB signaling, preventing its activation through the noncanonical pathway. A family of 16 compounds with this core has been synthesized and decorated with the aminopyrimidine ring. Affinity experiments have proved that some of these derivatives are able to bind NIK, a critical effector protein in the NF‐κB nonclassical inflammation pathway. The bioisosteric replacement of the aromatic rings in position 2 of the imidazolone core yields compounds with good affinity when a heteroatom is present. Indeed, compound **3d** was shown to inhibit in a dose‐dependent manner the nonclassical NF‐κB activation pathway triggered by TWEAK in cellular models, as assessed by NF‐κB2 p100 to p50 processing. Moreover, our computational framework lets us propose a theoretical binding mode that rationalizes the experimental affinities. Our results indicate that MD simulations coupled with binding affinity calculations with WaterSwap can provide a reliable assay to inform about drug binding affinity against NIK, for classification and hit prioritization.

Despite further extensive work that will be necessary to perfectly elucidate all possible off‐targets and mechanism of action elicited by these imidazolones, they represent a promising chemical tool for the study and modulation of the NF‐κB signaling in the context of kidney disease.

## 4 EXPERIMENTAL

### 4.1 Chemistry

#### 4.1.1 General

Oxygen‐ and moisture‐sensitive reactions were carried out under a nitrogen or argon atmosphere. Unless otherwise indicated, all chemicals and reagents were purchased from commercial suppliers (Merck, Across, Fischer…) and used without further purification. Reaction progress was monitored by analytical thin‐layer chromatography (TLC) using aluminum plates with silica Kieselgel 60 F254 of thickness 0.25 mm and visualized under 254 nm UV lamp. Flash column chromatography was carried out in the indicated solvent system using prepacked silica gel cartridges for use on the Biotage Purification System.

^1^H‐NMR spectra (see the Supporting Information) were recorded in CDCl_3_, CD_3_OD, or CD_3_SOCD_3_ solution on a Bruker 400 MHz, Varian 300, or 500 MHz spectrometer at 25°C and chemical shifts were recorded in parts per million (ppm). ^13^C‐NMR spectra were recorded at 100 MHz. Resonances are described using the following abbreviations: s (singlet), d (doublet), t (triplet), q (quartet), m (multiplet), br (broad), dd (doublet of doublets), and so on. Coupling constants (J) are reported in Hz and are rounded to the nearest 0.1 Hz. High‐performance liquid chromatography (HPLC) analysis of all final compounds was >95% pure. HPLC analysis was performed using a VARIANT HPLC and a KROMAPHASE C18 250 mm × 4.6 mm, 5 μm; elution was performed in gradient with formic acid 0.1% (v/v) in water and formic acid 0.1% (v/v) in acetonitrile.

The InChI codes of the investigated compounds, together with some biological activity data, are provided as Supporting Information.

#### 4.1.2 General procedure for the synthesis of amidine chlorides and 2‐bromo‐N‐(alkyl) acetamides

Suitable amidine chloride salts and 2‐bromo‐*N*‐(alkyl) acetamides were synthesized following the procedure described in the literature.^[^ ^20^, ^26^, ^27^ ^]^

#### 4.1.3 General procedure for the synthesis of compounds **1**

To a stirred solution of the corresponding aldehyde (1 equiv), the corresponding amidine hydrochloride (1.5 equiv), and dried NaHCO_3_ (5 equiv) in anhydrous *sec*‐butanol (2 mL/mmol), a solution of the corresponding 2‐bromo‐*N*‐(alkyl)‐acetamide (2.4 equiv) in *sec*‐butanol (1 mL/mmol) was added, and the reaction mixture was heated at reflux until the corresponding aldehyde could not be detected by TLC. Then, the reaction was cooled down to room temperature, the solid was filtered off, washed with EtOAc, and the solvent was concentrated under reduced pressure. The residue was purified by silica gel column chromatography to give the corresponding product.

Compounds **1a**, **1b**, and **1c** were synthesized as previously described by our group.^[^ ^20^, ^28^ ^]^

(*Z*)‐5‐[(1*H*‐Indol‐2‐yl)methylene]‐3‐(2‐methoxyethyl)‐2‐(pyridin‐4‐yl)‐3,5‐dihydro‐4*H*‐imidazol‐4‐one (**1d**): Obtained as a red oil (111.8 mg, 0.32 mmol, 10%) following the general procedure outlined above using 1*H*‐indole‐2‐carboxaldehyde (490 mg, 3.38 mmol) and the corresponding amidine chloride salt (800 mg, 5.08 mmol). R_*f*_ = 0.4 (CH_2_Cl_2_/MeOH 30:1). ^1^H‐NMR (300 MHz, CDCl_3_) *δ* 10.64 (br s, 1H, NH), 8.86 (d, *J* = 6.1 Hz, 2H, H‐2”, H‐6”), 7.87 (d, *J* = 6.1 Hz, 2H, H‐3”, H‐5”), 7.64 (d, *J* = 8.1 Hz, 1H, H‐4’), 7.45–7.33 (m, 2H, H‐7’, H‐6’), 7.29 (s, 1H, CH), 7.10 (t, *J* = 7.5 Hz, 1H, H‐5’), 7.03 (s, 1H, H‐3’), 3.93 (t, *J* = 5.1 Hz, 2H, N‐CH_2_), 3.65 (t, *J* = 5.1 Hz, 2H, CH_2_–O), 3.31 (s, 3H, CH_3_). ^13^C‐NMR (75 MHz, CDCl_3_) *δ* 170.0 (C), 158.6 (C), 150.1 (CH), 139.7 (C), 137.7 (C), 136.3 (C), 134.2 (C), 128.3 (C), 126.2 (CH), 123.3 (CH), 122.3 (CH), 120.9 (2 × CH), 120.4 (CH), 114.2 (CH), 111.9 (2 × CH), 69.9 (CH_2_), 59.3 (CH_3_), 42.6 (CH_2_). high‐resolution mass spectrometry (electrospray ionization ‐ time of flight (HRMS (ESI‐TOF)) calcd for C_20_H_19_N_4_O_2_ [M+H]^+^: 347.1503. Found: 347.1514.

(*Z*)‐5‐[(1*H*‐Indol‐2‐yl)methylene]‐2‐(furan‐2‐yl)‐3‐(2‐methoxyethyl)‐3,5‐dihydro‐4*H*‐imidazol‐4‐one (**1e**): Obtained as an orange oil (810 mg, 2.41 mmol, 57%) following the general procedure outlined above using 1*H*‐indole‐2‐carboxaldehyde (617 mg, 4.25 mmol) and the corresponding amidine chloride salt (934 mg, 6.38 mmol). R_*f*_ = 0.6 (CH_2_Cl_2_/EtOAc 4.5:1). ^1^H‐NMR (300 MHz, CDCl_3_) *δ* 10.75 (br s, 1H, NH), 7.74–7.70 (m, 1H, H‐6”), 7.64 (d, *J* = 7.4 Hz, 1H, H‐7’), 7.49 (d, *J* = 3.5 Hz, 1H, H‐3”), 7.44 (d, *J* = 8.4 Hz, 1H, H‐4’), 7.30 (t, *J* = 8.0 Hz, 1H, H‐6’), 7.25 (s, 1H, CH), 7.10 (t, *J* = 7.5 Hz, 1H, H‐5’), 6.97 (s, 1H, H‐3’), 6.69 (dd, *J* = 3.6, 1.7 Hz, 1H, H‐4”), 4.24 (t, *J* = 5.9 Hz, 2H, N‐CH_2_), 3.65 (t, *J* = 6.0 Hz, 2H, CH_2_–O), 3.35 (s, 3H, CH_3_–O). ^13^C‐NMR (75 MHz, CDCl_3_) *δ* 169.7 (C), 150.4 (C), 146.0 (CH), 144.5 (C), 139.3 (C), 136.7 (C), 134.8 (C), 128.4 (C), 125.5 (CH), 122.1 (CH), 120.6 (CH), 117.9 (CH), 116.6 (CH), 112.9 (CH), 112.8 (CH), 111.8 (CH), 70.7 (CH_2_), 59.3 (CH_3_), 41.6 (CH_2_). HRMS (ESI‐TOF) calcd for C_19_H_18_N_3_O_3_ [M+H]^+^: 336.1343. Found: 336.1347.

(*Z*)‐5‐[(1*H*‐Indol‐2‐yl)methylene]‐3‐(2‐methoxyethyl)‐2‐(thiophen‐2‐yl)‐3,5‐dihydro‐4*H*‐imidazol‐4‐one (**1f**): Obtained as a red oil (295.2 mg, 0.84 mmol, 41%) following the general procedure outlined above using 1*H*‐indole‐2‐carboxaldehyde (690 mg, 4.75 mmol) and the corresponding amidine chloride salt (1.2 g, 7.12 mmol). R_*f*_ = 0.3 (Hexane/EtOAc 2.5:1). ^1^H‐NMR (300 MHz, CDCl_3_) *δ* 10.79 (br s, 1H, NH), 7.97 (d, *J* = 3.8 Hz, 1H, H‐6”), 7.65 (m, 2H, H‐3”, H‐4”), 7.44 (d, *J* = 8.2 Hz, 1H, H‐4’), 7.35–7.20 (m, 3H, H‐7’, CH, H‐6’), 7.17–7.06 (m, 1H, H‐5’), 6.98 (s, 1H, H‐3’), 4.14 (t, *J* = 5.6 Hz, 2H, N‐CH_2_), 3.73 (t, *J* = 5.4 Hz, 2H, CH_2_‐O), 3.39 (s, 3H, CH_3_). ^13^C‐NMR (75 MHz, CDCl_3_) *δ* 170.0 (C), 154.6 (C), 139.3 (C), 136.5 (C), 134.8 (C), 132.2 (C), 131.1 (CH), 130.9 (CH), 128.7 (CH), 128.3 (C), 125.5 (CH), 122.1 (CH), 120.6 (CH), 117.7 (CH), 112.7 (CH), 111.8 (CH), 70.4 (CH_2_), 59.4 (CH_3_), 42.2 (CH_2_). HRMS (ESI‐TOF) calcd for C_19_H_18_N_3_O_2_S [M+H]^+^: 352.1114. Found: 352.1120.

(*Z*)‐5‐[(1*H*‐Indol‐2‐yl)methylene]‐2‐(furan‐3‐yl)‐3‐(2‐methoxyethyl)‐3,5‐dihydro‐4*H*‐imidazol‐4‐one (**1g**): Obtained as a red oil (326.7 mg, 0.97 mmol, 30%) following the general procedure outlined above using 1*H*‐indole‐2‐carboxaldehyde (465 mg, 3.20 mmol) and the corresponding amidine chloride salt (704 mg, 4.80 mmol). R_*f*_ = 0.4 (Hexane/EtOAc 3:1). ^1^H‐NMR (300 MHz, CDCl_3_) *δ* 10.77 (br s, 1H, NH), 8.33 (s, 1H, H‐2”), 7.68–7.56 (m, 2H, H‐4’, H‐6”), 7.41 (d, *J* = 8.3 Hz, 1H, H‐7’), 7.33–7.20 (m, 2H, H‐6’, CH), 7.15–7.05 (m, 2H, H‐5’, H‐4”), 6.95 (s, 1H, H‐3’), 3.95 (t, *J* = 5.3 Hz, 2H, N–CH_2_), 3.67 (t, *J* = 5.3 Hz, 2H, CH_2_–O), 3.34 (s, 3H, CH_3_). ^13^C‐NMR (75 MHz, CDCl_3_) *δ* 169.9 (C), 154.6 (C), 145.3 (CH), 143.8 (CH), 139.0 (C), 136.6 (C), 134.5 (C), 128.1 (C), 125.3 (CH), 121.8 (CH), 120.4 (CH), 117.4 (CH), 116.4 (C), 112.3 (CH), 111.6 (CH), 110.4 (CH), 70.3 (CH_2_), 59.1 (CH_3_), 41.9 (CH_2_). HRMS (ESI‐TOF) calcd for C_19_H_18_N_3_O_3_ [M+H]^+^: 336.1343. Found: 336.1354.

(*Z*)‐5‐[(1*H*‐Indol‐2‐yl)methylene]‐3‐(2‐methoxyethyl)‐2‐(thiophen‐3‐yl)‐3,5‐dihydro‐4*H*‐imidazol‐4‐one (**1h**): Obtained as a red oil (191 mg, 0.54 mmol, 18%) following the general procedure outlined above using 1*H*‐indole‐2‐carboxaldehyde (450 mg, 3.07 mmol) and the corresponding amidine chloride salt (749 mg, 4.60 mmol). R_*f*_ = 0.3 (Hexane/EtOAc 4:1). ^1^H‐NMR (300 MHz, CDCl_3_) *δ* 10.77 (br s, 1H, NH), 8.31 (d, *J* = 2.9 Hz, 1H, H‐2”), 7.76 (d, *J* = 5.1 Hz, 1H, H‐6”), 7.62 (d, *J* = 8.0 Hz, 1H, H‐4’), 7.49 (dd, *J* = 5.3, 3.2 Hz, 1H, H‐5”), 7.40 (d, *J* = 8.3 Hz, 1H, H‐7’), 7.29–7.24 (m, 2H, CH, H‐6’), 7.14–7.03 (m, 1H, H‐5’), 6.95 (s, 1H, H‐3’), 4.01 (t, *J* = 5.2 Hz, 2H, N‐CH_2_), 3.72 (t, *J* = 5.2 Hz, 2H, CH_2_–O), 3.36 (s, 3H, CH_3_). ^13^C‐NMR (75 MHz, CDCl_3_) *δ* 170.3 (C), 156.4 (C), 139.2 (C), 136.8 (C), 134.7 (C), 130.6 (C), 129.9 (CH), 128.6 (CH), 128.3 (C), 126.7 (CH), 125.5 (CH), 122.0 (CH), 120.6 (CH), 117.9 (CH), 112.6 (CH), 111.8 (CH), 70.5 (CH_2_), 59.4 (CH_3_), 42.5 (CH_2_). HRMS (ESI‐TOF) calcd for C_19_H_18_N_3_O_2_S [M+H]^+^: 352.1114. Found: 352.1119.

(*Z*)‐5‐[(1*H*‐Indol‐2‐yl)methylene]‐2‐(3‐chlorophenyl)‐3‐(2‐methoxyethyl)‐3,5‐dihydro‐4*H*‐imidazol‐4‐one (**1i**): Obtained as a red oil (589.9 mg, 1.55 mmol, 36%) following the general procedure outlined above using 1*H*‐indole‐2‐carboxaldehyde (633 mg, 4.36 mmol) and the corresponding amidine chloride salt (1.3 g, 6.54 mmol). R_*f*_ = 0.4 (CH_2_Cl_2_/EtOAc 2:1). ^1^H‐NMR (300 MHz, CDCl_3_) *δ* 10.67 (br s, 1H, NH), 7.93 (s, 1H, H‐2”), 7.82– 7.73 (m, 1H, H‐4”), 7.58 (d, *J* = 9.0 Hz, 1H, H‐4’), 7.51 (d, *J* = 8.0 Hz, 1H, H‐6”), 7.45 (t, *J* = 7.6 Hz, 1H, H‐5”), 7.37 (d, *J* = 8.3 Hz, 1H, H‐7’), 7.26 (s, 1H, CH), 7.23–7.20 (m, 1H, H‐6’), 7.10–6.99 (m, 1H, H‐5’), 6.94 (s, 1H, H‐3’), 3.86 (t, *J* = 5.2 Hz, 2H, N–CH_2_), 3.59 (t, *J* = 5.1 Hz, 2H, CH_2_–O), 3.24 (s, 3H, CH_3_). ^13^C‐NMR (75 MHz, CDCl_3_) *δ* 170.2 (C), 159.8 (C), 139.5 (C), 136.2 (C), 135.1 (C), 134.3 (C), 131.5 (CH), 131.3 (C), 130.3 (CH), 129.5 (CH), 128.3 (C), 127.4 (CH), 125.8 (CH), 122.1 (CH), 120.7 (CH), 119.2 (CH), 113.4 (CH), 111.9 (CH), 69.8 (CH_2_), 59.1 (CH_3_), 42.3 (CH_2_). HRMS (ESI‐TOF) calcd for C_21_H_19_ClN_3_O_2_ [M+H]^+^: 380.1160. Found: 380.1166.

(*Z*)‐5‐[(1*H*‐Indol‐2‐yl)methylene]‐2‐(4‐bromophenyl)‐3‐(2‐methoxyethyl)‐3,5‐dihydro‐4*H*‐imidazol‐4‐one (**1j**): Obtained as a red oil (805.8 mg, 1.90 mmol, 67%) following the general procedure outlined above using *1H*‐indole‐2‐carboxaldehyde (410 mg, 2.83 mmol) and the corresponding amidine chloride salt (1.0 g, 4.25 mmol). R_*f*_ = 0.4 (Hexane/EtOAc 2.5:1). ^1^H‐NMR (300 MHz, CDCl_3_) *δ* 10.68 (br s, 1H, NH), 7.80 (d, *J* = 8.6 Hz, 2H, H‐3”, H‐5”), 7.66 (d, *J* = 8.5 Hz, 2H, H‐2”, H‐6”), 7.59 (d, *J* = 8.0 Hz, 1H, H‐4’), 7.35 (d, *J* = 7.4 Hz, 1H, H‐7’), 7.28–7.17 (m, 2H, CH, H‐6’), 7.05 (t, *J* = 7.5 Hz, 1H, H‐5’), 6.94 (s, 1H, H‐3’), 3.87 (t, *J* = 5.3 Hz, 2H, N‐CH_2_), 3.60 (t, *J* = 5.3 Hz, 2H, CH_2_–O), 3.25 (s, 3H, CH_3_). ^13^C‐NMR (75 MHz, CDCl_3_) δ 170.3 (C), 160.2 (C), 139.3 (C), 136.5 (C), 134.4 (C), 132.2 (2 × CH), 130.8 (2 × CH), 128.4 (C), 128.2 (C), 126.2 (C), 125.6 (CH), 122.0 (CH), 120.6 (CH), 118.9 (CH), 113.1 (CH), 111.8 (CH), 69.7 (CH_2_), 59.1 (CH_3_), 42.3 (CH_2_). HRMS (ESI‐TOF) calcd for C_21_H_19_BrN_3_O_2_ [M+H]^+^: 424.0655. Found: 424.0668.

(*Z*)‐5‐[(1*H*‐Indol‐2‐yl)methylene]‐3‐(2‐methoxyethyl)‐2‐[4‐(trifluoromethyl)phenyl]‐3,5‐dihydro‐4*H*‐imidazol‐4‐one (**1k**): Obtained as a red oil (164.3 mg, 0.40 mmol, 53%) following the general procedure outlined above using 1*H*‐indole‐2‐carboxaldehyde (109 mg, 0.75 mmol) and the corresponding amidine chloride salt (253 mg, 1.12 mmol). R_*f*_ = 0.3 (Hexane/EtOAc 2.5:1). ^1^H‐NMR (300 MHz, CDCl_3_) *δ* 10.70 (br s, 1H, NH), 8.11 (d, *J* = 8.6 Hz, 2H, H‐3”, H‐5”), 7.84 (d, *J* = 8.3 Hz, 2H, H‐2”, H‐6”), 7.64 (d, *J* = 8.8 Hz, 1H, H‐4’), 7.40 (d, *J* = 8.3 Hz, 1H, H‐7’), 7.34 (s, 1H, CH), 7.29 (t, *J* = 8.1 Hz, 1H, H‐6’), 7.11 (d, *J* = 7.0 Hz, 1H, H‐5’), 7.02 (s, 1H, H‐3’), 3.92 (t, *J* = 5.2 Hz, 2H, N‐CH_2_), 3.66 (t, *J* = 5.2 Hz, 2H, CH_2_–O), 3.30 (s, 3H, CH_3_). ^13^C‐NMR (75 MHz, CDCl_3_) δ 170.0 (C), 159.7 (C), 139.4 (C), 136.1 (C), 134.1 (C), 132.9 (C), 132.9 (q, ^*2*^ *J*_*C‐F*_ = 31.5 Hz, C), (129.7 (2 × CH), 128.1 (C), 125.8 (q, ^*3*^ *J*_*C‐F*_ = 3.8 Hz) (CH), 125.7 (2 × CH, C), 122.0 (CH), 120.6 (CH), 119.4 (CH), 113.5 (CH), 112.4 (C), 111.7 (CH), 69.6 (CH_2_), 59.0 (CH_3_), 42.3 (CH_2_). ^19^F NMR (282 MHz, CDCl_3_) *δ* –62.98. HRMS (ESI‐TOF) calcd for C_22_H_19_F_3_N_3_O_2_ [M+H]^+^: 414.1424. Found: 414.1432.

(*Z*)‐5‐[(1*H*‐Indol‐2‐yl)methylene]‐2‐[4‐(dimethylamino)phenyl]‐3‐(2‐methoxyethyl)‐3,5‐dihydro‐4*H*‐imidazol‐4‐one (**1l**): Obtained as a red oil (151 mg, 0.39 mmol, 14%) following the general procedure outlined above using 1*H*‐indole‐2‐carboxaldehyde (410.8 mg, 2.83 mmol) and the corresponding amidine chloride salt (700 mg, 3.51 mmol). *R*_*f*_ = 0.3 (CH_2_Cl_2_/EtOAc 4:1). ^1^H‐NMR (300 MHz, CDCl_3_) *δ* 10.92 (br s, 1H, NH), 7.90 (d, *J* = 8.9 Hz, 2H, H‐3”, H‐5”), 7.61 (d, *J* = 7.2 Hz, 1H, H‐4’), 7.40 (d, *J* = 8.6 Hz, 1H, H‐7’), 7.23 (t, *J* = 8.1 Hz, 1H, H‐6’), 7.18 (s, 1H, CH), 7.07 (t, *J* = 7.5 Hz, 1H, H‐5’), 6.91 (s, 1H, H‐3’), 6.81 (d, *J* = 9.1 Hz, 2H, H‐2”, H‐6”), 4.02 (t, *J* = 5.9 Hz, 2H, N‐CH_2_), 3.68 (t, *J* = 5.7 Hz, 2H, CH_2_–O), 3.34 (s, 3H, CH_3_), 3.10 (s, 6H, 2 × CH_3_–N). ^13^C‐NMR (75 MHz, CDCl_3_) *δ* 170.9 (C), 161.1 (C), 152.5 (C), 139.0 (C), 137.0 (C), 135.1 (C), 130.6 (2 × CH), 128.4 (C), 125.0 (CH), 121.8 (CH), 120.4 (CH), 116.0 (CH), 115.7 (CH), 111.8 (2 × CH), 111.8 (CH), 111.5 (C), 70.1 (CH_2_), 59.4 (CH_3_), 42.5 (CH_2_), 40.4 (2 × CH_3_). HRMS (ESI‐TOF) calcd for C_23_H_25_N_4_O_2_ [M+H]^+^: 389.1972. Found: 389.1984.

(*Z*)‐5‐[(1*H*‐Indol‐2‐yl)methylene]‐3‐(2‐methoxyethyl)‐2‐(3‐methoxyphenyl)‐3,5‐dihydro‐4*H*‐imidazol‐4‐one (**1m**): Obtained as a red oil (763.2 mg, 2.03 mmol, 46%) following the general procedure outlined above using 1*H*‐indole‐2‐carboxaldehyde (639 mg, 4.40 mmol) and the corresponding amidine chloride salt (1.2 g, 6.59 mmol). R_*f*_ = 0.5 (CH_2_Cl_2_/EtOAc 2.5:1). ^1^H‐NMR (300 MHz, CDCl_3_) *δ* 10.79 (br s, 1H, NH), 7.64 (d, *J* = 8.0 Hz, 1H, H‐4’), 7.53 (s, 1H, H‐2”), 7.48 (d, *J* = 5.5 Hz, 2H, H‐4”, H‐6”), 7.40 (d, *J* = 8.3 Hz, 1H, H‐7’), 7.30 (s, 1H, CH), 7.26 (t, *J* = 7.6 Hz, 1H, H‐5”), 7.18–7.11 (m, 1H, H‐6’), 7.11–7.04 (m, 1H, H‐5’), 6.98 (s, 1H, H‐3’), 3.95 (t, *J* = 5.4 Hz, 2H, N‐CH_2_), 3.90 (s, 3H, CH_3_–O–Ar), 3.65 (t, *J* = 5.4 Hz, 2H, CH_2_–O), 3.30 (s, 3H, CH_3_–O). ^13^C‐NMR (75 MHz, CDCl_3_) *δ* 170.4 (C), 160.9 (C), 159.7 (C), 139.2 (C), 134.4 (C), 130.6 (C), 130.0 (CH), 128.1 (C), 125.4 (CH), 121.9 (CH), 121.3 (CH), 120.4 (CH), 118.5 (CH), 117.3 (CH), 114.9 (C), 114.3 (CH), 112.7 (CH), 111.7 (CH), 69.7 (CH_2_), 59.0 (CH_2_), 55.5 (CH_3_), 42.1 (CH_2_). HRMS (ESI‐TOF) calcd for C_22_H_22_N_3_O_3_ [M+H]^+^: 376.1656. Found: 376.1662.

(*Z*)‐5‐[(1*H*‐Indol‐2‐yl)methylene]‐3‐(2‐methoxyethyl)‐2‐(4‐methoxyphenyl)‐3,5‐dihydro‐4*H*‐imidazol‐4‐one (**1n**): Obtained as a red oil (332.2 mg, 0.88 mmol, 43%) following the general procedure outlined above using 1*H*‐indole‐2‐carboxaldehyde (300 mg, 2.05 mmol) and the corresponding amidine chloride salt (574 mg, 3.07 mmol). R_*f*_ = 0.3 (CH_2_Cl_2_/EtOAc 2.5:1). ^1^H‐NMR (300 MHz, CDCl_3_) *δ* 10.83 (br s, 1H, NH), 7.94 (d, *J* = 8.6 Hz, 2H, H‐3”, H‐5”), 7.63 (d, *J* = 8.1 Hz, 1H, H‐4’), 7.41 (d, *J* = 8.2 Hz, 1H, H‐7’), 7.26 (m, 2H, H‐6’, CH), 7.12–7.06 (m, 3H, CH, H‐2”, H‐6”, H‐5’), 6.96 (s, 1H, H‐3’), 3.98 (t, *J* = 5.7 Hz, 2H, N–CH_2_), 3.91 (s, 3H, CH_3_–O–Ar), 3.67 (t, *J* = 5.3 Hz, 2H, CH_2_–O), 3.31 (s, 3H, CH_3_–O). ^13^C‐NMR (75 MHz, CDCl_3_) *δ* 170.6 (C), 162.3 (C), 160.9 (C), 139.2 (C), 134.7 (C), 131.0 (2 × CH), 128.3 (C), 125.4 (CH), 122.0 (CH), 121.7 (C), 120.6 (CH), 117.7 (2 × CH), 114.7 (C), 114.5 (CH), 112.4 (CH), 111.8 (CH), 70.0 (CH_2_), 59.3 (CH_3_), 55.8 (CH_3_), 42.4 (CH_2_). HRMS (ESI‐TOF) calcd for C_22_H_22_N_3_O_3_ [M+H]^+^: 376.1656. Found: 376.1665.

(*Z*)‐5‐[(1*H*‐Indol‐2‐yl)methylene]‐2‐(4‐bromopyridin‐2‐yl)‐3‐(2‐methoxyethyl)‐3,5‐dihydro‐4*H*‐imidazol‐4‐one (**1o**): Obtained as a red oil (43.5 mg, 0.10 mmol, 24%) following the general procedure outlined above using 1*H*‐indole‐2‐carboxaldehyde (61.2 mg, 0.42 mmol) and the corresponding amidine chloride salt (150 mg, 0.63 mmol). R_*f*_ = 0.3 (CH_2_Cl_2_/EtOAc 2:1). ^1^H‐NMR (300 MHz, CDCl_3_) *δ* 10.60 (s, 1H, NH), 8.74 (s, 1H, H‐3”), 8.22 (d, *J* = 8.4 Hz, 1H, H‐6”), 8.02 (d, *J* = 6.5 Hz, 1H, H‐5”), 7.63 (d, *J* = 7.7 Hz, 1H, H‐4’), 7.42 (d, *J* = 7.5 Hz, 1H, H‐7’), 7.32–7.29 (m, 2H, CH, H‐6’), 7.15–7.05 (m, 1H, H‐5’), 6.99 (s, 1H, H‐3’), 4.50–4.44 (m, 2H, N–CH_2_), 3.62–3.56 (m, 2H, CH_2_–O), 3.26 (s, 3H, CH_3_). ^13^C‐NMR (75 MHz, CDCl_3_) *δ* 169.9 (C), 150.1 (CH), 147.3 (C), 145.4 (C), 139.6 (CH), 139.3 (C), 136.5 (C), 134.4 (C), 128.2 (C), 125.8 (CH), 125.5 (CH), 123.2 (C), 122.1 (CH), 120.6 (CH), 119.5 (CH), 113.7 (CH), 111.6 (CH), 70.8 (CH_2_), 58.9 (CH_3_), 41.6 (CH_2_). HRMS (ESI‐TOF) calcd for C_20_H_18_BrN_4_O_2_ [M+H]^+^: 425.0608. Found: 425.0605.

(*Z*)‐5‐[(1*H*‐Indol‐2‐yl)methylene]‐3‐(2‐methoxyethyl)‐2‐(1‐methyl‐1*H*‐indol‐3‐yl)‐3,5‐dihydro‐4*H*‐imidazol‐4‐one (**1p**): Obtained as a red oil (200.5 mg, 0.50 mmol, 27%) following the general procedure outlined above using 1*H*‐indole‐2‐carboxaldehyde (270 mg, 1.85 mmol) and the corresponding amidine chloride salt (582 mg, 2.77 mmol). R_*f*_ = 0.4 (CH_2_Cl_2_/EtOAc 1:1). ^1^H‐NMR (300 MHz, CDCl_3_) *δ* 11.20 (s, 1H, NH), 8.50–8.41 (m, 1H, H‐4”), 8.06 (s, 1H, H‐2”), 7.65 (d, *J* = 8.6 Hz, 1H, H‐4’), 7.45–7.33 (m, 4H, H‐7’, H‐7”, H‐6’, H‐6”), 7.30–7.24 (m, 1H, H‐5’), 7.19 (s, 1H, CH), 7.11 (t, *J* = 7.4 Hz, 1H, H‐5”), 6.92 (s, 1H, H‐3’), 4.05 (t, *J* = 5.3 Hz, 2H, N‐CH_2_), 3.88 (s, 3H, CH_3_‐N), 3.77 (t, *J* = 5.3 Hz, 2H, CH_2_–O), 3.41 (s, 3H, CH_3_–O). ^13^C‐NMR (75 MHz, CDCl_3_) *δ* 170.5 (C), 156.5 (C), 138.9 (C), 137.5 (C), 135.5 (C), 133.1 (CH), 128.5 (C), 127.5 (C), 124.9 (CH), 123.8 (CH), 122.2 (CH), 122.1 (CH, C), 121.8 (CH), 120.4 (CH), 114.9 (CH), 111.7 (CH), 110.7 (CH), 110.3 (CH), 104.4 (C), 70.9 (CH_2_), 59.6 (CH_3_), 42.4 (CH_2_), 34.0 (CH_3_). HRMS (ESI‐TOF) calcd for C_24_H_23_N_4_O_2_ [M+H]^+^: 399.1816. Found: 399.1822.

#### 4.1.4 General procedure for the synthesis of compounds **2**

To a stirred solution of compound **1** (1 equiv) in dry DMF:CH_2_Cl_2_ (25 mL, 1:2), *N*‐iodosuccinimide (NIS, 1 equiv) was added, and the mixture was stirred at room temperature until the starting material could not be detected by TLC. Then, the reaction mixture was washed with brine and the aqueous layer was extracted with EtOAc. The combined organic layer was dried over anhydrous Na_2_SO_4_ and concentrated under reduced pressure. The crude product was purified by silica gel column chromatography to give the corresponding halogenated product.

Compounds **2a**, **2b**, and **2c** were synthesized as previously described by our group.^[^ ^20^, ^28^ ^]^

(*Z*)‐5‐[(3‐Iodo‐1*H*‐indol‐2‐yl)methylene]‐3‐(2‐methoxyethyl)‐2‐(pyridin‐4‐yl)‐3,5‐dihydro‐4*H*‐imidazol‐4‐one (**2d**): Obtained as a red oil (84.6 mg, 0.18 mmol, 56%) following the general procedure outlined above using compound **1d** (111.8 mg, 0.32 mmol). R_*f*_ = 0.2 (CH_2_Cl_2_/EtOAc 1:2). ^1^H‐NMR (300 MHz, CDCl_3_) *δ* 11.04 (br s, 1H, NH), 8.85 (d, *J* = 6.0 Hz, 2H, H‐2”, H‐6”), 7.89 (d, *J* = 6.0 Hz, 2H, H‐3”, H‐5”), 7.52–7.41 (m, 2H, H‐4’, CH), 7.41–7.23 (m, 2H, H‐7’, H‐6’), 7.22–7.12 (m, 1H, H‐5’), 3.95 (t, *J* = 5.1 Hz, 2H, N‐CH_2_), 3.68 (t, *J* = 5.0 Hz, 2H, CH_2_–O), 3.31 (s, 3H, CH_3_). ^13^C‐NMR (75 MHz, CDCl_3_) *δ* 175.3 (C), 169.7 (C), 150.3 (C), 150.3 (CH), 139.0 (C), 137.3 (C), 135.4 (C), 131.2 (C), 127.0 (CH), 127.0 (CH), 123.1 (CH), 122.7 (2 × CH), 121.5 (CH), 119.0 (C), 112.0 (2 × CH), 69.7 (CH_2_), 59.2 (CH_3_), 42.5 (CH_2_). HRMS (ESI‐TOF) calcd for C_20_H_17_IN_4_O_2_ [M+H]^+^: 473.0469. Found: 473.0481.

(*Z*)‐2‐(Furan‐2‐yl)‐5‐[(3‐iodo‐1*H*‐indol‐2‐yl)methylene]‐3‐(2‐methoxyethyl)‐3,5‐dihydro‐4*H*‐imidazol‐4‐one (**2e**): Obtained as a red oil (755.1 mg, 1.64 mmol, 68%) following the general procedure outlined above using compound 1e (809.7 mg, 2.41 mmol). R_*f*_ = 0.3 (CH_2_Cl_2_/EtOAc 4:1). ^1^H‐NMR (300 MHz, CDCl_3_) *δ* 11.12 (br s, 1H, NH), 7.71 (s, 1H, H‐5”), 7.51–7.41 (m, 2H, H‐4’, H‐3”), 7.41–7.23 (m, 3H, H‐7’, H‐6’, CH), 7.19–7.09 (m, 1H, H‐5’), 6.72–6.64 (m, 1H, H‐4”), 4.22 (t, *J* = 5.9 Hz, 2H, N‐CH_2_), 3.64 (t, *J* = 5.8 Hz, 2H, CH_2_–O), 3.34 (s, 3H, CH_3_). ^13^C‐NMR (75 MHz, CDCl_3_) *δ* 169.5 (C), 160.2 (C), 151.1 (C), 146.3(CH), 144.4 (C), 138.8 (C), 138.0 (C), 136.1 (C), 131.3 (C), 126.5 (CH), 122.5 (CH), 121.4 (CH), 117.1 (CH), 116.6 (CH), 113.0 (CH), 112.0 (CH), 70.6 (CH_2_), 59.3 (CH_3_), 41.7 (CH_2_). HRMS (ESI‐TOF) calcd for C_19_H_17_IN_3_O_3_ [M+H]^+^: 462.0309. Found: 462.0313.

(*Z*)‐5‐[(3‐Iodo‐1*H*‐indol‐2‐yl)methylene]‐3‐(2‐methoxyethyl)‐2‐(thiophen‐2‐yl)‐3,5‐dihydro‐4*H*‐imidazol‐4‐one (**2f**): Obtained as a red oil (398.7 mg, 0.84 mmol, 98%) following the general procedure outlined above using compound **1f** (301 mg, 0.86 mmol). R_*f*_ = 0.6 (CH_2_Cl_2_/EtOAc 1:2). ^1^H‐NMR (300 MHz, CDCl_3_) *δ* 11.22 (s, 1H, NH), 8.01 (d, *J* = 3.9 Hz, 1H, H‐6”), 7.67 (d, *J* = 5.1 Hz, 1H, H‐3”), 7.47 (d, *J* = 8.1 Hz, 1H, H‐4’), 7.41–7.28 (m, 3H, CH, H‐4”, H‐7’), 7.27–7.11 (m, 2H, H‐6’, H‐5’), 4.15 (t, *J* = 5.5 Hz, 2H, N–CH_2_), 3.72 (t, *J* = 5.5 Hz, 2H, CH_2_–O), 3.36 (s, 3H, CH_3_). ^13^C‐NMR (75 MHz, CDCl_3_) *δ* 169.9 (C), 155.4 (C), 138.8 (C), 137.7 (C), 136.0 (C), 132.0 (C), 131.5 (CH), 131.3 (CH), 131.3 (CH), 128.8 (CH), 128.7 (C), 126.5 (CH), 122.5 (CH), 121.4 (CH), 116.4 (C), 112.0 (CH), 70.3 (CH_2_), 59.3 (CH_3_), 42.1 (CH_2_). HRMS (ESI‐TOF) calcd for C_19_H_17_IN_3_O_2_S [M+H]^+^: 478.0081. Found: 478.0086.

(*Z*)‐2‐(Furan‐3‐yl)‐5‐[(3‐iodo‐1*H*‐indol‐2‐yl)methylene]‐3‐(2‐methoxyethyl)‐3,5‐dihydro‐4*H*‐imidazol‐4‐one (**2g**): Obtained as a red oil (413.8 mg, 0.90 mmol, 92%) following the general procedure outlined above using compound **1g** (326.7 mg, 0.97 mmol). R_*f*_ = 0.3 (CH_2_Cl_2_/EtOAc 2.5:1). ^1^H‐NMR (300 MHz, CDCl_3_) *δ* 11.15 (br s, 1H, NH), 8.34 (s, 1H, H‐2”), 7.59 (s, 1H, H‐5”), 7.43 (d, *J* = 8.1 Hz, 1H, H‐4’), 7.38–7.30 (m, 1H, H‐7’), 7.32–7.28 (m, 1H, H‐6’), 7.25 (s, 1H, CH), 7.14 (t, *J* = 7.4 Hz, 1H, H‐5’), 7.08–7.05 (m, 1H, H‐4”), 3.96 (t, *J* = 5.2 Hz, 2H, N–CH_2_), 3.68 (t, *J* = 5.2 Hz, 2H, CH_2_–O), 3.35 (s, 3H, CH_3_). ^13^C‐NMR (75 MHz, CDCl_3_) *δ* 169.6 (C), 155.4 (C), 145.6 (CH), 143.9 (CH), 138.5 (C), 137.7 (C), 135.7 (C), 131.1 (C), 126.3 (CH), 122.3 (CH), 121.2 (CH), 116.3 (C), 116.2 (CH), 112.5 (C), 111.9 (CH), 110.4 (CH), 70.4 (CH_2_), 59.3 (CH_3_), 42.2 (CH_2_). HRMS (ESI‐TOF) calcd for C_19_H_17_F_3_IN_3_O_3_ [M+H]^+^: 462.0309. Found: 462.0308.

(*Z*)‐5‐[(3‐Iodo‐1*H*‐indol‐2‐yl)methylene]‐3‐(2‐methoxyethyl)‐2‐(thiophen‐3‐yl)‐3,5‐dihydro‐4*H*‐imidazol‐4‐one (**2h**): Obtained as a red oil (235.5 mg, 0.49 mmol, 91%) following the general procedure outlined above using compound **1h** (191 mg, 0.54 mmol). R_*f*_ = 0.4 (CH_2_Cl_2_/EtOAc 2:1). ^1^H‐NMR (300 MHz, CDCl_3_) *δ* 11.18 (br s, 1H, NH), 8.35 (s, 1H, H‐2”), 7.76 (d, *J* = 5.0 Hz, 1H, H‐5”), 7.54–7.47 (m, 1H, H‐4”), 7.44 (d, *J* = 7.9 Hz, 1H, H‐4’), 7.34 (m, 2H, H‐7’, H‐6’), 7.29 (s, 1H, CH), 7.20–7.09 (m, 1H, H‐5’), 4.00 (t, *J* = 5.1 Hz, 2H, N‐CH_2_), 3.71 (t, *J* = 5.1 Hz, 2H, CH_2_–O), 3.36 (s, 3H, CH_3_). ^13^C‐NMR (75 MHz, CDCl_3_) *δ* 170.0 (C), 157.0 (C), 138.5 (C), 137.8 (C), 135.8 (C), 131.1 (C), 130.2 (CH), 128.4 (CH), 126.7 (CH, C), 126.3 (CH, C), 122.3 (CH), 121.2 (CH), 116.4 (CH), 111.9 (CH), 70.2 (CH_2_), 59.2 (CH_3_), 42.2 (CH_2_). HRMS (ESI‐TOF) calcd for C_19_H_17_IN_3_O_2_S [M+H]^+^: 478.0081. Found: 478.0087.

(*Z*)‐2‐(3‐Chlorophenyl)‐5‐[(3‐iodo‐1*H*‐indol‐2‐yl)methylene]‐3‐(2‐methoxyethyl)‐3,5‐dihydro‐4*H*‐imidazol‐4‐one (**2i**): Obtained as a red oil (577.9 mg, 1.14 mmol, 74%) following the general procedure outlined above using compound **1i** (590 mg, 1.55 mmol). R_*f*_ = 0.4 (CH_2_Cl_2_/EtOAc 2.5:1). ^1^H‐NMR (300 MHz, CDCl_3_) *δ* 11.11 (br s, 1H, NH), 7.99 (t, *J* = 1.8 Hz, 1H, H‐2”), 7.84 (dt, *J* = 7.5, 1.4 Hz, 1H, H‐4”), 7.62–7.44 (m, 3H, H‐6”, H‐4’, H‐5”), 7.38 (m, 2H, CH, H‐7’), 7.34–7.28 (m, 1H, H‐6’), 7.17 (t, *J* = 7.5 Hz, 1H, H‐5’), 3.93 (t, *J* = 5.2 Hz, 2H, N–CH_2_), 3.66 (t, *J* = 5.2 Hz, 2H, CH_2_–O), 3.31 (s, 3H, CH_3_). ^13^C‐NMR (75 MHz, CDCl_3_) *δ* 170.1 (C), 160.6 (C), 138.9 (C), 137.7 (C), 135.6 (C), 135.1 (C), 131.6 (CH), 131.3 (C), 131.2 (C), 130.3 (CH, C), 129.4 (CH), 127.4 (CH), 126.7 (CH), 122.6 (CH), 121.5 (CH), 117.9 (CH), 112.2 (CH), 69.9 (CH_2_), 59.3 (CH_3_), 42.6 (CH_2_). HRMS (ESI‐TOF) calcd for C_21_H_17_ClIN_3_O_2_ [M+H]^+^: 506.0127. Found: 506.0127.

(*Z*)‐2‐(4‐Bromophenyl)‐5‐[(3‐iodo‐1*H*‐indol‐2‐yl)methylene]‐3‐(2‐methoxyethyl)‐3,5‐dihydro‐4*H*‐imidazol‐4‐one (**2j**): Obtained as a red oil (391.3 mg, 0.71 mmol, 37%) following the general procedure outlined above using compound **1j** (805 mg, 1.90 mmol). R_*f*_ = 0.3 (Hexane/EtOAc 3:1). ^1^H‐NMR (300 MHz, CDCl_3_) *δ* 11.15 (br s, 1H, NH), 7.86 (d, *J* = 8.6 Hz, 2H, H‐3”, H‐5”), 7.71 (d, *J* = 8.6 Hz, 2H, H‐2”, H‐6”), 7.47 (d, *J* = 8.1 Hz, 1H, H‐4’), 7.41–7.28 (m, 3H, CH, H‐7’, H‐6’), 7.17 (t, *J* = 7.3 Hz, 1H, H‐5’), 3.93 (t, *J* = 5.2 Hz, 2H, N‐CH_2_), 3.66 (t, *J* = 5.2 Hz, 2H, CH_2_–O), 3.30 (s, 3H, CH_3_). ^13^C‐NMR (75 MHz, CDCl_3_) *δ* 170.2 (C), 161.1 (C), 138.9 (C), 137.8 (C), 135.7 (C), 132.3 (2 × CH), 131.3 (C), 130.8 (2 × CH), 130.6 (C), 128.3 (C), 126.7 (CH), 126.5 (C), 122.6 (CH), 121.5 (CH), 117.7 (CH), 112.1 (CH), 69.9 (CH_2_), 59.3 (CH_3_), 42.6 (CH_2_). HRMS (ESI‐TOF) calcd for C_21_H_18_BrIN_3_O_2_ [M+H]^+^: 549.9622. Found: 549.9628.

(*Z*)‐5‐[(3‐Iodo‐1*H*‐indol‐2‐yl)methylene]‐3‐(2‐methoxyethyl)‐2‐[4‐(trifluoromethyl)phenyl]‐3,5‐dihydro‐4*H*‐imidazol‐4‐one (**2k**): Obtained as a red oil (190.6 mg, 0.35 mmol, 89%) following the general procedure outlined above using compound **1k** (164 mg, 0.40 mmol). R_*f*_ = 0.3 (CH_2_Cl_2_/EtOAc 2.5:1). ^1^H‐NMR (300 MHz, CDCl_3_) *δ* 11.06 (br s, 1H, NH), 8.09 (d, *J* = 8.2 Hz, 2H, H‐3”, H‐5”), 7.82 (d, *J* = 8.2 Hz, 2H, H‐2”, H‐6”), 7.44 (d, *J* = 8.0 Hz, 1H, H‐4’), 7.36 (s, 1H, CH), 7.34–7.25 (m, 2H, H‐7’, H‐6’), 7.14 (t, *J* = 7.1 Hz, 1H, H‐5’), 3.93 (t, *J* = 5.1 Hz, 2H, N–CH_2_), 3.67 (t, *J* = 5.1 Hz, 2H, CH_2_–O), 3.31 (s, 3H, CH_3_). ^13^C‐NMR (75 MHz, CDCl_3_) *δ* 170.1 (C), 160.7 (C), 138.9 (C), 137.6 (C), 135.6 (CH), 133.3 (q, ^*2*^ *J*_*C‐F*_ = 32.6 Hz, C), 132.9 (C), 131.2 (C), 129.8 (2 × CH), 126.8 (CH), 125.8 (q, ^*3*^ *J*_*C‐F*_ = 3.8 Hz, CH), 123.7 (q, ^*1*^ *J*_*C‐F*_ = 272.2 Hz, CF_3_), 122.6 (CH), 121.5 (CH), 118.3 (CH), 112.0 (2 × CH), 69.7 (CH_2_), 59.1 (CH_3_), 42.5 (CH_2_). ^19^F NMR (282 MHz, CDCl_3_) *δ* –62.95. HRMS (ESI‐TOF) calcd for C_22_H_18_F_3_IN_3_O_2_ [M+H]^+^: 540.0390. Found: 540.0397.

(*Z*)‐2‐[4‐(Dimethylamino)phenyl]‐5‐[(3‐iodo‐1*H*‐indol‐2‐yl)methylene]‐3‐(2‐methoxyethyl)‐3,5‐dihydro‐4*H*‐imidazol‐4‐one (**2l**): Obtained as a red oil (120.9 mg, 0.24 mmol, 60%) following the general procedure outlined above using compound **1l** (151 mg, 0.39 mmol). R_*f*_ = 0.3 (Hexane/EtOAc 2.5:1). ^1^H‐NMR (300 MHz, CDCl_3_) *δ* 11.38 (br s, 1H, NH), 7.91 (d, *J* = 9.1 Hz, 2H, H‐3”, H‐5”), 7.45 (d, *J* = 7.9 Hz, 1H, H‐4’), 7.38–7.25 (m, 3H, H‐7’, CH, H‐6’), 7.20– 7.09 (m, 1H, H‐5’), 6.80 (d, *J* = 9.1 Hz, 2H, H‐2”, H‐6”), 4.03 (t, *J* = 5.7 Hz, 2H, N‐CH_2_), 3.69 (t, *J* = 5.7 Hz, 2H, CH_2_‐O), 3.34 (s, 3H, CH_3_), 3.10 (s, 6H, 2 x CH_3_‐N). ^13^C‐NMR (75 MHz, CDCl_3_) δ 170.9 (C), 161.9 (C), 152.6 (C), 138.5 (C), 136.4 (C), 131.3 (C), 130.6 (2 x CH), 130.3 (C), 125.9 (CH), 124.6 (C), 122.2 (CH), 121.2 (CH), 115.6 (C), 114.5 (CH), 112.0 (CH), 111.7 (2 x CH), 70.1 (CH_2_), 59.4 (CH_2_), 42.5 (2 x CH_3_), 40.4 (CH_2_). HRMS (ESI‐TOF) calcd for C_23_H_24_IN_4_O_2_ [M+H]^+^: 515.0938. Found: 515.0946.

(*Z*)‐5‐[(3‐Iodo‐1*H*‐indol‐2‐yl)methylene]‐3‐(2‐methoxyethyl)‐2‐(3‐methoxyphenyl)‐3,5‐dihydro‐4*H*‐imidazol‐4‐one (**2m**): Obtained as a red oil (635.5 mg, 1.27 mmol, 62%) following the general procedure outlined above using compound **1m** (763 mg, 2.03 mmol). R_*f*_ = 0.4 (CH_2_Cl_2_/EtOAc 2:1). ^1^H‐NMR (300 MHz, CDCl_3_) *δ* 11.18 (br s, 1H, NH), 7.53 (s, 1H, H‐2”), 7.50–7.39 (m, 3H, H‐4”, H‐4’, H‐6”), 7.37–7.21 (m, 3H, CH, H‐5”, H‐7’), 7.13 (t, *J* = 6.7 Hz, 2H, H‐6’, H‐5’), 3.94 (t, *J* = 5.1 Hz, 2H, N–CH_2_), 3.89 (s, 3H, CH_3_–O–Ar), 3.65 (t, *J* = 5.0 Hz, 2H, CH_2_–O), 3.31 (s, 3H, CH_3_). ^13^C‐NMR (75 MHz, CDCl_3_) *δ* 170.3 (C), 161.9 (C), 159.9 (C), 138.8 (C), 138.1 (C), 135.8 (C), 131.2 (C), 130.6 (C), 130.2 (CH, C), 126.5 (CH), 122.5 (CH), 121.5 (CH), 121.4 (CH), 117.6 (CH), 117.3 (CH), 114.6 (CH), 112.2 (CH), 70.0 (CH_2_), 59.3 (CH_3_), 55.9 (CH_3_), 42.5 (CH_2_). HRMS (ESI‐TOF) calcd for C_22_H_21_IN_3_O_3_ [M+H]^+^: 502.0622. Found: 502.0628.

(*Z*)‐5‐[(3‐Iodo‐1*H*‐indol‐2‐yl)methylene]‐3‐(2‐methoxyethyl)‐2‐(4‐methoxyphenyl)‐3,5‐dihydro‐4*H*‐imidazol‐4‐one (**2n**): Obtained as a red oil (420.2 mg, 0.84 mmol, 95%) following the general procedure outlined above using compound **1n** (332.2 mg, 0.88 mmol). R_*f*_ = 0.3 (CH_2_Cl_2_/EtOAc 2:1). ^1^H‐NMR (300 MHz, CDCl_3_) *δ* 11.26 (s, 1H, NH), 7.94 (d, *J* = 9.0 Hz, 2H, H‐3”, H‐5”), 7.46 (d, *J* = 8.6 Hz, 1H, H‐4’), 7.39–7.25 (m, 3H, H‐7’, H‐6’, CH), 7.16 (dd, *J* = 8.0, 6.8, 1H, H‐5’), 7.08 (d, *J* = 9.0 Hz, 2H, H‐2”, H‐6”), 3.97 (t, *J* = 5.4 Hz, 2H, N‐CH_2_), 3.92 (s, 3H, CH_3_–O–Ar), 3.66 (t, *J* = 5.5 Hz, 2H, CH_2_–O), 3.31 (s, 3H, CH_3_). ^13^C‐NMR (75 MHz, CDCl_3_) *δ* 170.5 (C), 162.4 (C), 161.7 (C), 138.6 (C), 138.3 (C), 136.0 (C), 131.2 (C), 131.0 (2 × CH), 130.7 (C), 126.3 (CH), 122.4 (CH), 121.5 (C), 121.3 (CH), 116.2 (2 × CH), 114.5 (CH), 112.1 (CH), 70.0 (CH_2_), 59.3 (CH_3_), 55.8 (CH_3_), 42.5 (CH_2_). HRMS (ESI‐TOF) calcd for C_22_H_21_F_3_IN_3_O_3_ [M+H]^+^: 502.0622. Found: 502.0630.

(*Z*)‐2‐(4‐Bromopyridin‐2‐yl)‐5‐[(3‐iodo‐1*H*‐indol‐2‐yl)methylene]‐3‐(2‐methoxyethyl)‐3,5‐dihydro‐4*H*‐imidazol‐4‐one (**2o**): Obtained as a red oil (40.3 mg, 0.07 mmol, 71%) following the general procedure outlined above using compound **1o** (43.5 mg, 0.10 mmol). R_*f*_ = 0.3 (CH_2_Cl_2_/EtOAc 3:1). ^1^H‐NMR (300 MHz, CDCl_3_) *δ* 11.04 (s, 1H, NH), 8.78 (d, *J* = 2.3 Hz, 1H, H‐3”), 8.24 (d, *J* = 8.5 Hz, 1H, H‐6”), 8.07 (dd, *J* = 8.5, 2.3 Hz, 1H, H‐5”), 7.48 (d, *J* = 7.6 Hz, 1H, H‐4’), 7.44–7.31 (m, 3H, H‐7’, CH, H‐6’), 7.18 (t, *J* = 7.4 Hz, 1H, H‐5’), 4.50 (t, *J* = 5.8 Hz, 2H, N–CH_2_), 3.60 (t, *J* = 5.8 Hz, 2H, CH_2_–O), 3.27 (s, 3H, CH_3_). ^13^C‐NMR (75 MHz, CDCl_3_) *δ* 170.0 (C), 157.7 (C), 150.4 (CH), 147.3 (C), 139.9 (CH), 139.0 (C), 137.8 (C), 135.8 (C), 131.4 (C), 127.0 (CH), 125.8 (CH), 123.6 (C), 122.8 (CH), 121.6 (CH), 119.8 (C), 118.6 (CH), 112.0 (CH), 70.9 (CH_2_), 59.1 (CH_3_), 41.8 (CH_2_). HRMS (ESI‐TOF) calcd for C_20_H_17_BrIN_4_O_2_ [M+H]^+^: 550.9574. Found: 550.9572.

(*Z*)‐5‐[(3‐Iodo‐1*H*‐indol‐2‐yl)methylene]‐3‐(2‐methoxyethyl)‐2‐(1‐methyl‐1*H*‐indol‐3‐yl)‐3,5‐dihydro‐4*H*‐imidazol‐4‐one (**2p**): Obtained as a red oil (25.7 mg, 0.05 mmol, 33%) following the general procedure outlined above using compound **1p** (58 mg, 0.15 mmol). R_*f*_ = 0.3 (CH_2_Cl_2_/EtOAc 1:1). ^1^H‐NMR (300 MHz, CDCl_3_) *δ* 11.62 (br s, 1H, NH), 8.46–8.37 (m, 1H, H‐7’), 8.14 (s, 1H, H‐2”), 7.51–7.36 (m, 4H, H‐7”, H‐4’, H‐4”, H‐6’), 7.32–7.27 (m, 2H, H‐6”, H‐5”), 7.23 (s, 1H, CH), 7.17 (t, *J* = 8.0 Hz, 1H, H‐5”), 4.08 (t, *J* = 5.3 Hz, 2H, N–CH_2_), 3.93 (s, 3H, N–CH_3_), 3.80 (t, *J* = 5.3 Hz, 2H, CH_2_–O), 3.42 (s, 3H, CH_3_–O). ^13^C‐NMR (75 MHz, CDCl_3_) *δ* 170.3 (C), 157.3 (C), 138.5 (C), 137.6 (C), 136.7 (C), 133.6 (C), 131.3 (C), 127.4 (C), 125.9 (CH), 123.9 (CH), 122.4 (CH), 122.2 (CH, C), 122.0 (CH), 121.2 (CH), 113.7 (CH), 112.6 (C), 111.9 (CH), 110.4 (CH), 104.2 (C), 70.9 (CH_2_), 59.6 (CH_3_), 42.6 (CH_2_), 34.2 (CH_3_). HRMS (ESI‐TOF) calcd for C_24_H_21_IN_4_O_2_ [M+H]^+^: 525.0782. Found: 525.0791.

#### 4.1.5 General procedure for the synthesis of final products **3**

In a microwave vial, it was added compound **2** (1 equiv), K_2_CO_3_ (1.4 equiv), Pd(PPh_3_)_4_ (5 mol%), and the corresponding pinacol boronate (1.4 equiv), and the mixture was suspended in degassed toluene/MeOH (4:1) (4 mL/mmol). The vial was tightly sealed and the suspension was irradiated for 20 min at a preselected temperature of 120°C (microwave assisted). After the reaction finished, the crude mixture was added to water and extracted with EtOAc. The combined organic layer was dried over anhydrous Na_2_SO_4_ and concentrated under reduced pressure. The crude product was purified by silica gel column chromatography to give the corresponding product.

(*Z*)‐5‐{[3‐(2‐Aminopyrimidin‐5‐yl)‐1*H*‐indol‐2‐yl]methylene}‐3‐(2‐methoxyethyl)‐2‐phenyl‐3,5‐dihydro‐4*H*‐imidazol‐4‐one (**3a**): Obtained as an orange solid (71.5 mg, 0.16 mmol, 38%) following the general procedure outlined above using compound **2a** (200 mg, 0.42 mmol) and 2‐aminopyrimidine‐5‐boronic acid pinacol ester (131.3 mg, 0.59 mmol). R_*f*_ = 0.3 (EtOAc). Mp 206°C (decomp.). ^1^H‐NMR (300 MHz, CDCl_3_) *δ* 11.15 (br s, 1H, NH), 8.52 (s, 2H, H‐4’, H‐6’), 7.94 (dd, *J* = 7.4, 2.1 Hz, 2H, H‐2”’, H‐6”’), 7.63–7.58 (m, 3H, H‐3”’, H‐5”’, H‐4”), 7.45 (d, *J* = 8.3 Hz, 1H, H‐7”), 7.33 (t, *J* = 7.6 Hz, 1H, H‐6”), 7.22 (s, 1H, CH), 7.20–7.09 (m, 1H, H‐5”), 5.35 (br s, 2H, NH_2_), 3.96 (t, *J* = 5.4 Hz, 2H, N–CH_2_), 3.62 (t, *J* = 5.4 Hz, 2H, CH_2_–O), 3.27 (s, 3H, CH_3_). ^13^C‐NMR (75 MHz, CDCl_3_) *δ* 170.1 (C), 161.5 (C), 161.3 (C), 158.6 (2 × CH), 138.4 (C), 137.6 (C), 131.9 (C), 131.4 (CH, C), 129.4 (C), 129.0 (2 × CH), 128.9 (2 × CH), 126.9 (C), 126.2 (CH), 121.1 (CH), 120.0 (CH), 118.1 (C), 115.5 (CH), 111.9 (CH), 69.5 (CH_2_), 58.9 (CH_3_), 42.0 (CH_2_). HRMS (ESI‐TOF) calcd for C_25_H_23_N_6_O_2_ [M+H]^+^: 439.1877. Found: 439.1887.

(*Z*)‐5‐{[3‐(2‐Aminopyrimidin‐5‐yl)‐1*H*‐indol‐2‐yl]methylene}‐3‐(2‐methoxyethyl)‐3,5‐dihydro‐4*H*‐imidazol‐4‐one (**3b**): Obtained as an orange solid (21.2 mg, 0.06 mmol, 23%) following the general procedure outlined above using compound **2b** (100 mg, 0.25 mmol) and 2‐aminopyrimidine‐5‐boronic acid pinacol ester (78.3 mg, 0.35 mmol). R_*f*_ = 0.3 (CH_2_Cl_2_/MeOH 20:1). Mp 203°C (decomp.). ^1^H‐NMR (300 MHz, CDCl_3_) *δ* 11.03 (br s, 1H, NH), 8.48 (s, 2H, H‐6’, H‐4’), 7.85 (s, 1H, H‐2), 7.60 (d, *J* = 8.1 Hz, 1H, H‐4”), 7.44 (d, *J* = 9.1 Hz, 1H, H‐7”), 7.34 (t, *J* = 7.6 Hz, 1H, H‐6”), 7.19–7.12 (m, 2H, CH, H‐5”), 5.31 (br s, 2H, NH_2_), 3.79 (t, *J* = 4.7 Hz, 2H, N–CH_2_), 3.55 (t, *J* = 4.7 Hz, 2H, CH_2_–O), 3.37 (s, 3H, CH_3_). ^13^C‐NMR (75 MHz, CDCl_3_) *δ* 168.2 (C), 161.9 (C), 158.8 (2 × CH), 152.4 (CH), 138.5 (C), 137.0 (C), 131.3 (C), 126.9 (C), 126.3 (CH), 126.3 (C), 121.0 (CH), 120.2 (CH), 117.9 (C), 117.1 (CH), 111.8 (CH), 70.6 (CH_2_), 59.0 (CH_3_), 41.3 (CH_2_). HRMS (ESI‐TOF) calcd for C_19_H_19_N_6_O_2_ [M+H]^+^: 363.1564. Found: 363.1569.

(*Z*)‐5‐{[3‐(2‐Aminopyrimidin‐5‐yl)‐1*H*‐indol‐2‐yl]methylene}‐3‐(2‐methoxyethyl)‐2‐methyl‐3,5‐dihydro‐4*H*‐imidazol‐4‐one (**3c**): Obtained as a yellow solid (136.9 mg, 0.36 mmol, 74%) following the general procedure outlined above using compound **2c** (200 mg, 0.49 mmol) and 2‐aminopyrimidine‐5‐boronic acid pinacol ester (151.3 mg, 0.68 mmol). R_*f*_ = 0.2 (EtOAc). Mp 205°C (decomp.). ^1^H‐NMR (300 MHz, CDCl_3_) *δ* 11.11 (br, s, 1H, NH), 8.48 (s, 2H, H‐4’, H‐6’), 7.65–7.56 (m, 1H, H‐4”), 7.46 (d, *J* = 9.0 Hz, 1H, H‐7”), 7.34 (t, *J* = 8.1 Hz, 1H, H‐6”), 7.13 (t, *J* = 7.8 Hz, 1H, H‐5”), 7.08 (s, 1H, CH), 5.43 (br s, 2H, NH_2_), 3.79 (t, *J* = 4.5 Hz, 2H, N‐CH_2_), 3.54 (t, *J* = 4.4 Hz, 2H, CH_2_–O), 3.32 (s, 3H, CH_3_–O), 2.47 (s, 3H. CH_3_). ^13^C‐NMR (75 MHz, CDCl_3_) *δ* 169.1 (C), 162.0 (C), 161.7 (C), 158.7 (2 × CH), 138.1 (C), 137.2 (C), 131.6 (C), 126.9 (C), 125.8 (CH), 120.8 (CH), 120.0 (CH), 118.8 (C), 117.9 (C), 114.3 (CH), 111.8 (CH), 70.6 (CH_2_), 59.1 (CH_3_), 41.2 (CH_2_), 16.1 (CH_3_). HRMS (ESI‐TOF) calcd for C_20_H_21_N_6_O_2_ [M+H]^+^: 377.1721. Found: 377.1727.

(*Z*)‐5‐{[3‐(2‐Aminopyrimidin‐5‐yl)‐1*H*‐indol‐2‐yl]methylene}‐3‐(2‐methoxyethyl)‐2‐(pyridin‐4‐yl)‐3,5‐dihydro‐4*H*‐imidazol‐4‐one (**3d**): Obtained as a red solid (17.0 mg, 0.04 mmol, 22%) following the general procedure outlined above using compound **2d** (84.6 mg, 0.18 mmol) and 2‐aminopyrimidine‐5‐boronic acid pinacol ester (55.4 mg, 0.25 mmol). R_*f*_ = 0.4 (CH_2_Cl_2_/MeOH 20:1). Mp 149–151°C. ^1^H‐NMR (300 MHz, CDCl_3_) *δ* 10.98 (br s, 1H, NH), 8.87 (d, *J* = 6.1 Hz, 2H, H‐2”’, H‐6”’), 8.52 (s, 2H, H‐4’, H‐6’), 7.90 (d, *J* = 6.1 Hz, 2H, H‐3”’, H‐6”’), 7.62 (d, *J* = 7.8 Hz, 1H, H‐4”), 7.46 (d, *J* = 7.5 Hz, 1H, H‐7”), 7.40–7.33 (m, 1H, H‐6”), 7.28 (s, 1H, CH), 7.16 (t, *J* = 8.0 Hz, 1H, H‐5”), 5.51 (br s, 2H, NH_2_), 3.94 (t, *J* = 5.1 Hz, 2H, N–CH_2_), 3.65 (t, *J* = 5.0 Hz, 2H, CH_2_–O), 3.30 (s, 3H, CH_3_). ^13^C‐NMR (75 MHz, CDCl_3_) *δ* 169.7 (C), 162.1 (C), 158.9 (2 × CH), 150.6 (2 × CH), 138.9 (C), 137.2 (C), 137.0 (C), 131.7 (C), 128.6 (C), 127.1 (C), 126.9 (CH), 123.1 (2 x CH), 121.4 (CH), 121.2 (C), 120.6 (CH), 118.0 (C), 117.6 (CH), 112.2 (CH), 69.9 (CH_2_), 59.3 (CH_3_), 42.6 (CH_2_). HRMS (ESI‐TOF) calcd for C_24_H_22_N_7_O_2_ [M+H]^+^: 440.1829. Found: 440.1839.

(*Z*)‐5‐{[3‐(2‐Aminopyrimidin‐5‐yl)‐1*H*‐indol‐2‐yl]methylene}‐2‐(furan‐2‐yl)‐3‐(2‐methoxyethyl)‐3,5‐dihydro‐4*H*‐imidazol‐4‐one (**3e**): Obtained as a red solid (17.0 mg, 0.04 mmol, 18%) following the general procedure outlined above using compound **2e** (100 mg, 0.22 mmol) and 2‐aminopyrimidine‐5‐boronic acid pinacol ester (67.1 mg, 0.30 mmol). R_*f*_ = 0.2 (CH_2_Cl_2_/EtOAc 1:4). Mp 215°C (decomp.). ^1^H‐NMR (300 MHz, CDCl_3_) *δ* 11.08 (br s, 1H, H‐1”), 8.50 (br s, 2H, H‐4’, H‐6’), 7.73 (d, *J* = 1.7 Hz, 1H, H‐6”’), 7.62 (d, *J* = 8.0 Hz, 1H, H‐4”), 7.54–7.43 (m, 2H, H‐3”’, H‐7”), 7.35 (t, *J* = 7.6 Hz, 1H, H‐6”), 7.21–7.09 (m, 2H, CH, H‐5”), 6.70 (dd, *J* = 3.6, 1.8 Hz, 1H, H‐4”’), 5.29 (s, 2H, NH_2_), 4.22 (t, *J* = 6.5 Hz, 2H, CH_2_–N), 3.63 (t, *J* = 5.8 Hz, 2H, CH_2_–O), 3.33 (s, 3H, CH_3_). ^13^C‐NMR (75 MHz, CDCl_3_) *δ* 169.4 (C), 161.8 (C), 158.8 (2 x CH), 150.7 (C), 146.2 (CH), 144.4 (C), 138.6 (C), 137.6 (C), 132.3 (CH), 127.2 (C), 126.3 (CH), 121.2 (CH), 120.3 (CH), 118.3 (C), 116.9 (CH), 115.1 (CH), 113.0 (C), 112.9 (C), 112.0 (CH), 70.6 (CH_2_), 59.3 (CH_3_), 41.7 (CH_2_). HRMS (ESI‐TOF) calcd for C_23_H_21_N_6_O_3_ [M+H]^+^: 429.1670. Found: 429.1668.

(*Z*)‐5‐{[3‐(2‐Aminopyrimidin‐5‐yl)‐1*H*‐indol‐2‐yl]methylene}‐3‐(2‐methoxyethyl)‐2‐(thiophen‐2‐yl)‐3,5‐dihydro‐4*H*‐imidazol‐4‐one (**3f**): Obtained as a red solid (54.6 mg, 0.12 mmol, 59%) following the general procedure outlined above using compound **2f** (100 mg, 0.21 mmol) and 2‐aminopyrimidine‐5‐boronic acid pinacol ester (64.8 mg, 0.29 mmol). R_*f*_ = 0.4 (CH_2_Cl_2_/MeOH 20:1). Mp 220°C (decomp.). ^1^H‐NMR (300 MHz, CDCl_3_) *δ* 11.08 (s, 1H, NH), 8.49 (s, 2H, H‐4’, H‐6’), 7.96 (d, *J* = 4.3 Hz, 1H, H‐6”’), 7.62 (m, 2H, H‐3”’, H‐4”), 7.44 (d, *J* = 8.2 Hz, 1H, H‐7”), 7.38–7.10 (m, 4H, H‐4”’, H‐5”, H‐6”, CH), 5.42 (s, 2H, NH_2_), 4.10 (t, *J* = 5.5 Hz, 2H, NH‐CH_2_), 3.69 (t, *J* = 5.0 Hz, 2H, CH_2_–O), 3.35 (s, 3H, CH_3_). ^13^C‐NMR (75 MHz, CDCl_3_) *δ* 169.7 (C), 162.1 (C), 158.9 (2 × CH), 154.8 (C), 138.6 (C), 137.3 (C), 132.2 (C), 132.1 (C), 131.3 (CH), 131.1 (CH), 128.8 (CH), 127.1 (C), 126.3 (CH), 121.2 (CH), 120.3 (CH), 119.8 (C), 118.2 (C), 115.0 (CH), 112.0 (CH), 70.4 (CH_2_), 59.4 (CH_3_), 42.2 (CH_2_). HRMS (ESI‐TOF) calcd for C_23_H_20_N_6_O_2_SNa [M+Na]^+^: 467.1261. Found: 467.1248.

(*Z*)‐5‐{[3‐(2‐Aminopyrimidin‐5‐yl)‐1*H*‐indol‐2‐yl]methylene}‐2‐(furan‐3‐yl)‐3‐(2‐methoxyethyl)‐3,5‐dihydro‐4*H*‐imidazol‐4‐one (**3g**): Obtained as a red solid (28.3 mg, 0.07 mmol, 30%) following the general procedure outlined above using compound **2g** (100 mg, 0.22 mmol) and 2‐aminopyrimidine‐5‐boronic acid pinacol ester (67.1 mg, 0.30 mmol). R_*f*_ = 0.4 (CH_2_Cl_2_/MeOH 20:1). Mp 215°C (decomp.). ^1^H‐NMR (300 MHz, CDCl_3_) *δ* 11.16 (br s, 1H, NH), 8.53 (s, 2H, H‐4’, H‐6’), 8.38 (s, 1H, H‐2”’), 7.62–7.59 (m, 2H, H‐6”’, H‐4”), 7.48 (d, *J* = 8.8 Hz, 1H, H‐4”’), 7.42–7.30 (m, 1H, H‐7”), 7.19–7.17 (m, 1H, H‐6”), 7.14–7.11 (m, 2H, CH, H‐5”), 5.66 (br, s, 2H, NH_2_), 3.99 (t, *J* = 5.1 Hz, 2H, N–CH_2_), 3.69 (t, *J* = 5.2 Hz, 2H, CH_2_–O), 3.35 (s, 3H, CH_3_).^13^C‐NMR (75 MHz, CDCl_3_) *δ* 169.7 (C), 162.0 (C), 159.1 (C), 158.8 (2 × CH), 154.9 (C), 145.5 (CH), 143.9 (CH), 138.3 (C), 137.3 (C), 131.9 (C), 127.0 (C), 126.0 (CH), 121.0 (CH), 120.1 (CH), 118.0 (C), 116.3 (C), 114.9 (CH), 111.8 (CH), 110.4 (CH), 70.3 (CH_2_), 59.1 (CH_3_), 42.0 (CH_2_). HRMS (ESI‐TOF) calcd for C_23_H_21_N_6_O_3_ [M+H]^+^: 429.1670. Found: 429.1683.

(*Z*)‐5‐{[3‐(2‐Aminopyrimidin‐5‐yl)‐1*H*‐indol‐2‐yl]methylene}‐3‐(2‐methoxyethyl)‐2‐(thiophen‐3‐yl)‐3,5‐dihydro‐4*H*‐imidazol‐4‐one (**3h**): Obtained as a red solid (63.2 mg, 0.14 mmol, 68%) following the general procedure outlined above using compound **2h** (100 mg, 0.21 mmol) and 2‐aminopyrimidine‐5‐boronic acid pinacol ester (64.8 mg, 0.29 mmol). R_*f*_ = 0.4 (CH_2_Cl_2_/MeOH 20:1). Mp 216°C (decomp.). ^1^H‐NMR (300 MHz, CDCl_3_) *δ* 11.14 (br s, 1H, NH), 8.51 (s, 2H, H‐4’, H‐6’), 8.36 (s, 1H, H‐2”’), 7.79 (d, *J* = 4.8 Hz, 1H, H‐6”’), 7.62 (d, *J* = 8.1 Hz, 1H, H‐4”), 7.56–7.44 (m, 1H, H‐4”’), 7.47–7.45 (m, 1H, H‐7”), 7.34 (t, *J* = 7.6 Hz, 1H, H‐6”), 7.23–7.09 (m, 2H, CH, H‐5”), 5.30 (br s, 2H, NH_2_), 4.03 (t, *J* = 5.1 Hz, 2H, N‐CH_2_), 3.72 (t, *J* = 5.1 Hz, 2H, CH_2_–O), 3.36 (s, 3H, CH_3_). ^13^C‐NMR (75 MHz, CDCl_3_) *δ* 170.0 (C), 161.2 (C), 158.5 (2 x CH), 156.7 (C), 138.3 (C), 137.5 (C), 132.0 (C), 130.2 (C), 130.1 (CH), 128.4 (CH), 126.9 (C), 126.7 (CH), 126.1 (CH), 121.1 (CH), 120.0 (CH), 118.8 (C), 118.1 (C), 114.8 (CH), 111.9 (CH), 70.1 (CH_2_), 59.1 (CH_3_), 42.3 (CH_2_). HRMS (ESI‐TOF) calcd for C_23_H_21_N_6_O_2_ [M+H]^+^: 445.1441. Found: 445.1454.

(*Z*)‐5‐{[3‐(2‐Aminopyrimidin‐5‐yl)‐1*H*‐indol‐2‐yl]methylene}‐2‐(3‐chlorophenyl)‐3‐(2‐methoxyethyl)‐3,5‐dihydro‐4*H*‐imidazol‐4‐one (**3i**): Obtained as a red solid (21.5 mg, 0.05 mmol, 23%) following the general procedure outlined above using compound **2i** (100 mg, 0.20 mmol) and 2‐aminopyrimidine‐5‐boronic acid pinacol ester (61.2 mg, 0.28 mmol). R_*f*_ = 0.4 (CH_2_Cl_2_/MeOH 20:1). Mp 207°C (decomp.). ^1^H‐NMR (300 MHz, CDCl_3_) *δ* 11.05 (br s, 1H, NH), 8.52 (s, 2H, H‐4’, H‐6’), 8.00 (s, 1H, H‐2”’), 7.85 (d, *J* = 7.4 Hz, 1H, H‐6”’), 7.64–7.56 (m, 2H, H‐4”, H‐4”’), 7.55–7.45 (m, 2H, H‐7”, H‐5”’), 7.41–7.30 (m, 1H, H‐6”), 7.23 (s, 1H, CH), 7.16 (t, *J* = 7.8 Hz, 1H, H‐5”), 5.37 (br s, 2H, NH_2_), 3.93 (t, *J* = 5.2 Hz, 2H, N–CH_2_), 3.64 (t, *J* = 5.2 Hz, 2H, CH_2_–O), 3.30 (s, 3H, CH_3_). ^13^C‐NMR (75 MHz, CDCl_3_) *δ* 169.9 (C), 161.9 (C), 160.0 (C), 158.8 (2 × CH), 138.6 (C), 137.2 (C), 135.0 (C), 131.7 (C), 131.4 (CH), 131.2 (C), 130.1 (CH, C), 129.3 (CH), 127.2 (CH), 126.9 (C), 126.4 (CH), 121.1 (CH), 120.2 (CH), 117.9 (C), 116.4 (CH), 112.0 (CH), 69.6 (CH_2_), 59.0 (CH_3_), 42.2 (CH_2_). HRMS (ESI‐TOF) calcd for C_25_H_22_ClN_6_O_2_ [M+H]^+^: 473.1487. Found: 473.1493.

(*Z*)‐5‐{[3‐(2‐Aminopyrimidin‐5‐yl)‐1*H*‐indol‐2‐yl]methylene}‐2‐(4‐bromophenyl)‐3‐(2‐methoxyethyl)‐3,5‐dihydro‐4*H*‐imidazol‐4‐one (**3j**): Obtained as an orange solid (46.1 mg, 0.09 mmol, 49%) following the general procedure outlined above using compound **2j** (100 mg, 0.18 mmol) and 2‐aminopyrimidine‐5‐boronic acid pinacol ester (56.3 mg, 0.25 mmol). R_*f*_ = 0.3 (EtOAc/MeOH 9:1). Mp 230°C (decomp.). ^1^H‐NMR (300 MHz, CDCl_3_) *δ* 11.06 (br s, 1H, NH), 8.51 (s, 2H, H‐4’, H‐6’), 7.87 (d, *J* = 6.9 Hz, 2H, H‐2”’, H‐6”’), 7.73 (d, *J* = 6.8 Hz, 2H, H‐3”’, H‐5”’), 7.62 (d, *J* = 8.1 Hz, 1H, H‐4”), 7.45 (d, *J* = 8.8 Hz, 1H, H‐7”), 7.35 (t, *J* = 7.0 Hz, 1H, H‐6”), 7.22 (s, 1H, CH), 7.17–7.13 (t, *J* = 7.5 Hz, 1H, H‐5”), 5.26 (s, 2H, NH_2_), 3.92 (t, *J* = 5.5 Hz, 2H, N–CH_2_), 3.64 (t, *J* = 5.1 Hz, 2H, CH_2_–O), 3.29 (s, 3H, CH_3_). ^13^C‐NMR (75 MHz, CDCl_3_) *δ* 170.0 (C), 161.5 (C), 160.5 (C), 158.6 (2 x CH), 138.4 (C), 137.4 (C), 132.2 (2 × CH), 131.8 (C), 130.6 (2 × CH), 128.2 (C), 126.9 (C), 126.3 (CH), 126.2 (C), 121.1 (CH), 120.1 (CH), 119.6 (C), 118.0 (C), 115.9 (CH), 111.9 (CH), 69.6 (CH_2_), 59.0 (CH_3_), 42.3 (CH_2_). HRMS (ESI‐TOF) calcd for C_25_H_22_BrN_6_O_2_ [M+H]^+^: 517.0982. Found: 517.0995.

(*Z*)‐5‐{[3‐(2‐Aminopyrimidin‐5‐yl)‐1*H*‐indol‐2‐yl]methylene}‐3‐(2‐methoxyethyl)‐2‐[4‐(trifluoromethyl)phenyl]‐3,5‐dihydro‐4*H*‐imidazol‐4‐one (**3k**): Obtained as a red solid (22.6 mg, 0.04 mmol, 24%) following the general procedure outlined above using compound **2k** (100 mg, 0.19 mmol) and 2‐aminopyrimidine‐5‐boronic acid pinacol ester (57.4 mg, 0.26 mmol). R_*f*_ = 0.3 (CH_2_Cl_2_/EtOAc 1:9). Mp 221°C (decomp.). ^1^H‐NMR (500 MHz, CDCl_3_) *δ* 11.03 (br s, 1H, NH), 8.52 (s, 2H, H‐4’, H‐6’), 8.12 (d, *J* = 8.1 Hz, 2H, H‐3”’, H‐5”’), 7.85 (d, *J* = 8.1 Hz, 2H, H‐2”’, H‐6”’), 7.62 (d, *J* = 8.6 Hz, 1H, H‐4”), 7.44 (d, *J* = 8.2 Hz, 1H, H‐7”), 7.40–7.29 (m, 1H, H‐6”), 7.27 (s, 1H, CH), 7.15 (t, *J* = 8.0 Hz, 1H, H‐5”), 5.30 (br s, 2H, NH_2_), 3.93 (t, *J* = 5.1 Hz, 2H, N–CH_2_), 3.65 (t, *J* = 5.1 Hz, 2H, CH_2_–O), 3.30 (s, 3H, CH_3_). ^13^C‐NMR (126 MHz, CDCl_3_) *δ* 169.8 (C), 162.1 (C), 160.0 (C), 158.9 (2 × CH), 138.6 (C), 137.1 (C), 133.4 (q, ^*2*^ *J*_*C‐F*_ = 32.8 Hz, CH), 132.9 (q, ^*4*^ *J*_*C‐F*_ = 1.3 Hz, C), 131.6 (C), 129.6 (2 × CH), 126.9 (C), 126.5 (CH), 125.8 (q, ^*3*^ *J*_*C‐F*_ = 3.8 Hz, CH), 125.8 (q, ^*1*^ *J*_*C‐F*_ = 272.7 Hz, CF_3_), 121.1 (CH), 120.6 (C), 120.3 (CH), 117.9 (C), 116.8 (CH), 111.9 (CH), 110.0 (C), 69.6 (CH_2_), 59.0 (CH_3_), 42.3 (CH_2_). ^19^F NMR (282 MHz, CDCl_3_) *δ* –62.99. HRMS (ESI‐TOF) calcd for C_26_H_22_F_3_N_6_O_2_ [M+H]^+^: 507.1751. Found: 507.1763.

(*Z*)‐5‐{[3‐(2‐Aminopyrimidin‐5‐yl)‐1*H*‐indol‐2‐yl]methylene}‐2‐[4‐(dimethylamino)phenyl]‐3‐(2‐methoxyethyl)‐3,5‐dihydro‐4*H*‐imidazol‐4‐one (**3l**): Obtained as a red solid (39.0 mg, 0.08 mmol, 69%) following the general procedure outlined above using compound **2l** (60.0 mg, 0.12 mmol) and 2‐aminopyrimidine‐5‐boronic acid pinacol ester (36.1 mg, 0.16 mmol). R_*f*_ = 0.4 (CH_2_Cl_2_/EtOAc 2.5:1). Mp 207°C (decomp.). ^1^H‐NMR (300 MHz, CDCl_3_) *δ* 11.53 (br s, 1H, NH), 8.53 (br s, 2H, H‐4’, H‐6’), 7.94 (d, *J* = 8.9 Hz, 2H, H‐2”’, H‐6”’), 7.61 (d, *J* = 8.6 Hz, 1H, H‐4”), 7.46 (d, *J* = 7.9 Hz, 1H, H‐7”), 7.32 (t, *J* = 7.7 Hz, 1H, H‐6”), 7.12 (m, 2H, H‐5”, CH), 6.83 (d, *J* = 9.1 Hz, 2H, H‐3”’, H‐5”’), 5.54 (s, 2H, NH_2_), 4.03 (t, *J* = 5.8 Hz, 2H, N‐CH_2_), 3.69 (t, *J* = 5.7 Hz, 2H, CH_2_–O), 3.34 (s, 3H, CH_3_–O), 3.12 (s, 6H, 2 × CH_3_–N). ^13^C‐NMR (101 MHz, CDCl_3_) *δ* 170.9 (C), 162.1 (C), 161.5 (C), 159.0 (2 x CH), 152.5 (C), 138.4 (C), 138.2 (C), 132.6 (C), 130.6 (2 x CH), 127.2 (C), 125.6 (CH), 120.9 (CH), 120.0 (CH), 118.5 (C),i 118.4 (C), 115.8 (C), 113.2 (CH), 111.9 (CH), 111.7 (2 × CH), 70.0 (CH_2_), 59.2 (CH_3_), 42.3 (CH_2_), 40.3 (2 × CH_3_). HRMS (ESI‐TOF) calcd for C_27_H_28_N_7_O_2_ [M+H]^+^: 482.2299. Found: 482.2305.

(*Z*)‐5‐{[3‐(2‐Aminopyrimidin‐5‐yl)‐1*H*‐indol‐2‐yl]methylene}‐3‐(2‐methoxyethyl)‐2‐(3‐methoxyphenyl)‐3,5‐dihydro‐4*H*‐imidazol‐4‐one (**3m**): Obtained as a red solid (23.7 mg, 0.05 mmol, 25%) following the general procedure outlined above using compound **2m** (100 mg, 0.20 mmol) and 2‐aminopyrimidine‐5‐boronic acid pinacol ester (61.7 mg, 0.28 mmol). R_*f*_ = 0.3 (CH_2_Cl_2_/MeOH 20:1). Mp 210°C (decomp.). ^1^H‐NMR (300 MHz, CDCl_3_) *δ* 11.12 (br s, 1H, NH), 8.51 (s, 2H, H‐4’, H‐6’), 7.66–7.39 (m, 5H, H‐2”’, H‐4”, H‐6”’, H‐7”, H‐4”’), 7.33 (t, *J* = 7.6 Hz, 1H, H‐6”), 7.22 (s, 1H, CH), 7.14 (t, *J* = 6.7 Hz, 2H, H‐5”’, H‐5”), 5.36 (br s, 2H, NH_2_), 3.93 (m, 5H, N–CH_2_, Ar–O–CH_3_), 3.64 (t, *J* = 5.2 Hz, 2H, CH_2_–O), 3.29 (s, 3H, CH_3_). ^13^C‐NMR (75 MHz, CDCl_3_) *δ* 170.5 (C), 162.3 (C), 161.3 (C), 159.9 (C), 159.2 (C), 159.0 (2 × CH), 138.6 (C), 137.6 (C), 132.0 (C), 130.7 (C), 130.2 (CH), 127.1 (C), 126.3 (CH), 121.5 (CH), 121.1 (CH), 120.3 (CH), 118.2 (CH), 117.5 (C), 116.0 (CH), 114.5 (CH), 112.1 (CH), 69.9 (CH_2_), 59.3 (CH_3_), 55.9 (CH_3_), 42.5 (CH_2_). HRMS (ESI‐TOF) calcd for C_26_H_25_N_6_O_3_ [M+H]^+^: 469.1983. Found: 469.1996.

(*Z*)‐5‐{[3‐(2‐Aminopyrimidin‐5‐yl)‐1*H*‐indol‐2‐yl]methylene}‐3‐(2‐methoxyethyl)‐2‐(4‐methoxyphenyl)‐3,5‐dihydro‐4*H*‐imidazol‐4‐one (**3n**): Obtained as a red solid (9.5 mg, 0.02 mmol, 10%) following the general procedure outlined above using compound **2n** (100 mg, 0.20 mmol) and 2‐aminopyrimidine‐5‐boronic acid pinacol ester (61.7 mg, 0.28 mmol). R_*f*_ = 0.2 (Hexane/EtOAc 1:9). Mp 209°C (decomp.). ^1^H‐NMR (300 MHz, CDCl_3_) *δ* 11.17 (br s, 1H, NH), 8.51 (s, 2H, H‐4’, H‐6’), 7.95 (d, *J* = 8.8 Hz, 2H, H‐2”’, H‐6”’), 7.62 (d, *J* = 8.1 Hz, 1H, H‐4”), 7.45–7.42 (m, 1H, H‐7”), 7.32 (t, *J* = 7.5 Hz, 1H, H‐6”), 7.22–7.04 (m, 4H, CH, H‐5”, H‐3”’, H‐6”’), 5.43 (br s, 2H, NH_2_), 3.96 (d, *J* = 5.3 Hz, 2H, N–CH_2_), 3.92 (s, 3H, CH_3_–O–Ar), 3.65 (t, *J* = 5.4 Hz, 2H, CH_2_–O), 3.30 (s, 3H, CH_3_). ^13^C‐NMR (75 MHz, CDCl_3_) *δ* 170.5 (C), 162.3 (C), 162.1 (C), 161.1 (C), 159.0 (2 × CH), 138.4 (C), 137.8 (C), 132.1 (C), 130.9 (2 × CH), 127.1 (C), 126.0 (CH), 121.6 (C), 121.0 (CH), 120.2 (CH), 119.4 (C), 118.1 (C), 114.9 (CH), 114.5 (2 × CH), 111.9 (CH), 69.8 (CH_2_), 59.1 (CH_3_), 55.6 (CH_3_), 42.2 (CH_2_). HRMS (ESI‐TOF) calcd for C_26_H_25_N_6_O_3_ [M+H]^+^: 469.1983. Found: 469.1996.

(*Z*)‐5‐{[3‐(2‐Aminopyrimidin‐5‐yl)‐1*H*‐indol‐2‐yl]methylene}‐2‐(4‐bromopyridin‐2‐yl)‐3‐(2‐methoxyethyl)‐3,5‐dihydro‐4*H*‐imidazol‐4‐one (**3o**): Obtained as a red solid (8.5 mg, 0.02 mmol, 22%) following the general procedure outlined above using compound **2o** (41 mg, 0.07 mmol) and 2‐aminopyrimidine‐5‐boronic acid pinacol ester (23.0 mg, 0.10 mmol). R_*f*_ = 0.3 (EtOAc). Mp 155–157°C. ^1^H‐NMR (500 MHz, CDCl_3_) *δ* 11.00 (s, 1H, NH), 8.80 (s, 1H, H‐3”’), 8.53 (s, 2H, H‐4’, H‐6’), 8.29 (d, *J* = 8.4 Hz, 1H, H‐5”’), 8.10 (d, *J* = 6.6 Hz, 1H, H‐6”’), 7.63 (d, *J* = 8.1 Hz, 1H, H‐4”), 7.49 (d, *J* = 8.2 Hz, 1H, H‐7”), 7.39 (t, *J* = 7.6 Hz, 1H, H‐6”), 7.27 (s, 1H, CH), 7.18 (t, *J* = 7.5 Hz, 1H, H‐5”), 5.55 (s, 2H, NH_2_), 4.51 (t, *J* = 5.8 Hz, 2H, N–CH_2_), 3.60 (t, *J* = 5.8 Hz, 2H, CH_2_–O), 3.26 (s, 3H, CH_3_). ^13^C‐NMR (101 MHz, CDCl_3_) *δ* 175.4 (C), 169.8 (C), 161.6 (C), 158.6 (2 × CH), 157.1 (C), 150.2 (CH, C), 147.3 (C), 139.7 (CH), 138.6 (C), 131.8 (C), 127.0 (C), 126.6 (CH), 125.5 (CH), 123.3 (C), 121.2 (CH), 120.3 (CH), 117.7 (C), 116.8 (CH), 111.8 (CH), 70.6 (CH_2_), 58.7 (CH_3_), 41.5 (CH_2_). HRMS (ESI‐TOF) calcd for C_24_H_21_BrN_7_O_2_ [M+H]^+^: 518.0935. Found: 518.0945.

(*Z*)‐5‐{[3‐(2‐Aminopyrimidin‐5‐yl)‐1*H*‐indol‐2‐yl]methylene}‐3‐(2‐methoxyethyl)‐2‐(1‐methyl‐1*H*‐indol‐3‐yl)‐3,5‐dihydro‐4*H*‐imidazol‐4‐one (**3p**): Obtained as a red solid (25.7 mg, 0.05 mmol, 47%) following the general procedure outlined above using compound **2p** (58 mg, 0.11 mmol) and 2‐aminopyrimidine‐5‐boronic acid pinacol ester (34.2 mg, 0.15 mmol). R_*f*_ = 0.3 (CH_2_Cl_2_/MeOH 40:1). Mp 201°C (decomp.). ^1^H‐NMR (400 MHz, DMSO) *δ* 11.44 (br s, 1H, NH), 8.50 (d, *J* = 7.7 Hz, 1H, H‐4”’), 8.47 (s, 2H, H‐4’, H‐6’), 8.41 (s, 1H, H‐2”’), 7.69 (d, *J* = 7.4 Hz 1H, H‐4”’), 7.66–7.56 (m, 2H, H‐4”, H‐7”’), 7.50–7.42 (m, 2H, H‐7”, H‐5”’), 7.34 (t, *J* = 7.5 Hz, 1H, H‐6”), 7.14 (t, *J* = 7.4 Hz, 1H, H‐5”), 6.91 (br s, 2H, NH_2_), 6.85 (s, 1H, CH), 4.17 (m, 2H, N–CH_2_), 3.98 (s, 3H, N–CH_3_), 3.63 (m, 2H, CH_2_–O), 3.24 (s, 3H, O–CH_3_). ^13^C NMR (101 MHz, DMSO) *δ* 169.7 (C), 163.1 (C), 158.7 (2 × CH), 157.2 (C), 138.4 (C), 138.2 (C), 137.5 (C), 134.8 (C), 131.9 (C), 127.0 (CH), 126.9 (C), 125.7 (CH), 123.7 (C), 122.6 (C), 122.2 (CH), 121.1 (CH), 120.1 (CH), 118.0 (CH), 116.4 (CH), 112.5 (CH), 111.5 (CH), 110.5 (C), 103.3 (CH), 70.0 (CH_2_), 58.8 (CH_3_), 55.4 (CH_2_), 33.9 (CH_3_). HRMS (ESI‐TOF) calcd for C_28_H_26_N_7_O_2_ [M+H]^+^: 492.2142. Found: 492.2148.

### 4.2 Pharmacological assays

#### 4.2.1 NIK binding assays

Affinity constants were determined by purchasing the KINOME*scan*™ Profiling Service from Eurofins Discovery, using a protocol that measures the ability of a compound to compete with an immobilized, active site‐directed ligand that binds to the protein of interest. Briefly, kinase‐tagged T7 phage strains were prepared in an *Escherichia coli* host derived from the BL21 strain, then were grown to log‐phase and infected with T7 phage and incubated with shaking at 32°C until lysis. Streptavidin‐coated magnetic beads were treated with biotinylated small molecule ligands for 30 min at room temperature to generate affinity resins for kinase assays. The liganded beads were blocked with excess biotin and washed with blocking buffer (SeaBlock (Pierce), 1% bovine serum albumin, 0.05% Tween 20, 1 mM dithiothreitol [DTT]) to remove unbound ligand and to reduce nonspecific binding. Binding reactions were assembled by combining kinases, liganded affinity beads, and test compounds in 1× binding buffer (20% SeaBlock, 0.17× phosphate‐buffered saline (PBS), 0.05% Tween 20, 6 mM DTT). The assay plates were incubated at room temperature with shaking for 1 h and the affinity beads were washed with wash buffer (1× PBS, 0.05% Tween 20). The beads were then resuspended in elution buffer (1× PBS, 0.05% Tween 20, 0.5 μM nonbiotinylated affinity ligand) and incubated at room temperature with shaking for 30 min. The kinase concentration in the eluates was measured by quantitative polymerase chain reaction (qPCR). Binding constants (Kds) were calculated with a standard dose–response curve using the Hill equation (The Hill Slope was set to –1) and data from two independent experiments.

#### 4.2.2 MTT viability assay

Human fibroblasts (HFF‐1) were acquired from the American Type Culture Collection and the culture media was purchased from Merck: DMEM + 10% fetal bovine serum (FBS) + 10% antibiotic (penicillin/streptomycin/amphotericin B). The cells were treated with ligands **3b**–**3 h** at 10 μM, freshly prepared using the culture medium. Quadruplicate experiments were run. For MTT viability assays, after 24 h incubation, 50 μL of MTT (5 mg/mL) was added, and an additional incubation of 4 h was allowed. The medium in each well was then replaced by DMSO, and the absorbance was measured at 570 nm using an enzyme‐linked immunosorbent assay (ELISA) plate reader. Absorbances were normalized using negative controls (untreated cells, in quadruplicate, with a maximum of 0.25% DMSO content).

#### 4.2.3 Western blot

Medium‐chain triglycerides (MCT) cells are a cultured line of kidney proximal tubular epithelial cells harvested originally from the renal cortex of SJL mice and have been extensively characterized.^[^ ^29^ ^]^ MCT cells were cultured in roswell park memorial institute 1640 (GIBCO), 10% decomplemented fetal bovine serum (FBS), 2 mM glutamine, 100 U/mL penicillin, and 100 µg/mL streptomycin, in 5% CO2 at 37°C.^[^ ^29^ ^]^ Recombinant human soluble TWEAK (Millipore) was used at 100 ng/mL based on prior dose–response experiments.^[^ ^30^, ^31^, ^32^ ^]^

Cell samples were homogenized in lysis buffer (50 mM TrisHCl, 150 mM NaCl, 2 mM ethylenediaminetetraacetic acid, 2 mM ethylene glycol tetraacetic acid, 0.2% Triton X‐100, 0.3% NP‐40, 0.1 mM phenylmethylsulfonyl fluoride, and 1 µg/mL pepstatin A) and separated by 10% sodium dodecyl sulfate‐polyacrylamide gel (SDS‐PAGE) under reducing conditions. After electrophoresis, samples were transferred to PVDF membranes (Millipore), blocked with 5% skimmed milk in PBS/0.5% v/v Tween 20 for 1 h, washed with PBS/Tween, and incubated with NF‐κB2 p100/p52 (1:100, Cell Signaling) antibody diluted in 5% milk PBS/Tween as previously described.^[^ ^30^ ^]^ Blots were washed with PBS/Tween, incubated with appropriate horseradish peroxidase‐conjugated secondary antibody (1:5000, GEHealthcare), developed with the chemiluminescence method (ECL) (Fisher Scientific), and probed with mouse monoclonal anti‐α‐tubulin (1:10000, Sigma) or anti‐GAPDH (1:5000, Millipore) antibodies. Levels of expression were corrected for minor differences in loading.

### 4.3 Molecular modeling

#### 4.3.1 Protein preparation

Since all crystallographic structures of human NIK deposited in the Protein Data Base (PDB) lack point amino acids or short loops, homology modeling techniques were chosen to generate a full model of the protein. To this aim, the SWISS‐MODEL webserver was used by retrieving NIK UniProt sequence (ID: Q99558) and as a template the PDB: 6Z1Q (DFG‐in conformation), maintaining the inhibitor bound to the protein. Residue protonation states and orientations from the final model (QMEAN0 = –0.21) were assigned using the H++ webserver (http://newbiophysics.cs.vt.edu/H++/).

#### 4.3.2 Molecular docking

Ligands and their physicochemical parameters were obtained from SMILES codes with RDKit as previously described elsewhere.^[^ ^33^ ^]^ Protonation states at pH 7.4 were explored using MarvinSketch 24.1.0 software (https://chemaxon.com). Docking calculations were performed using GOLD software,^[^ ^34^ ^]^ by defining a 7.5 Å radius sphere around the co‐crystalized ligand present in the homology model. The genetic algorithm combined with the ChemPLP scoring function was selected for ligand conformational sampling and scoring. The maximum number of poses per ligand was set to 5, while the rest of the docking parameters were kept as default. Docking results were evaluated through visual inspection and docking score and the docking protocol was validated by redocking the cocrystalized ligand (Q58) onto the receptor. All figures were rendered with PyMol 2.6 (The PyMOL Molecular Graphics System, Version 2.0 Schrödinger, LLC.) or VMD 1.9.4a.^[^ ^35^ ^]^

#### 4.3.3 Molecular dynamics simulations

Protein–ligand complexes simulations were prepared with the CHARMM‐GUI webserver, using the coordinates generated from docking calculations for all‐atom classical MD.^[^ ^36^ ^]^ Protein was described with the CHARMM36 forcefield, while ligand charges and parameters were derived from CGenFF (2.5.1 version).^[^ ^37^, ^38^ ^]^ Complexes were immersed in cubic TIP3P water molecules ensuring a minimum distance of 10 Å from the complex to the box edges, then K^+^ (68) Cl^–^ (64) ions were added to a final concentration of 0.15 mM till neutralization.^[^ ^39^ ^]^ Using CHARMM‐GUI scripts, all systems were minimized, equilibrated, and simulated for 250 ns at 298 K with Gromacs 2023.1 in the NPT ensemble using the Verlet scheme and the Nose‐Hoover thermostat.^[^ ^40^ ^]^ The LINCS algorithm was used to constrain all bonds involving hydrogen atoms, to use a 2 fs time step throughout the simulation.^[^ ^41^ ^]^ Simulations were performed on Windows 11 workstations equipped with an NVIDA RTX A2000 12GB GPU yielding a performance of 89 ns/day for an approximately 74,000 atoms system.

WaterSwap simulations were performed using the python code implemented in Sire suite from BioSimSpace.^[^ ^42^ ^]^ The WaterSwap method calculates the absolute protein–ligand binding free energy using an explicit water model by swapping the ligand with an equal volume of water inside the binding site.^[^ ^24^ ^]^ Using a single Monte Carlo simulation to calculate the WaterSwap reaction coordinate, this method avoids the cavitation problems inherent to implicit solvent methods used in similar calculations.^[^ ^43^ ^]^ To do these calculations, Amber ff14SB forcefield was chosen to describe protein parameters while GAFF2 and AM1‐BCC charges were selected to parameterize ligands.^[^ ^44^, ^45^ ^]^ WaterSwap calculations were performed for 1000 iterations using 25 million moves of Monte Carlo sampling across each of the 16 *λ* windows set as default. Free energy of binding was evaluated using three statistical approaches: free energy perturbation, thermodynamic integration, and Bennett's acceptance ratio with an agreement between values below 1 kcal/mol.

## CONFLICT OF INTEREST STATEMENT

The authors declare no conflicts of interest.

## Supporting information

Supporting information.

Supporting information.

## Data Availability

The data that support the findings of this study are available from the corresponding author upon reasonable request.
