# Supplementary material for: Identification and study of new NF‐κB‐inducing kinase ligands derived from the imidazolone scaffold
Source: Arch Pharm (Weinheim). 2024 Nov 27;358(1):e2400614. doi: 10.1002/ardp.202400614 (PMC11704032; doi:10.1002/ardp.202400614)
Supplement: Supplementary file 1 — Supporting information. [file ARDP-358-e2400614-s002.docx]

**Supplemental Material: Novel Compounds and Biological Screening Results**

Identification and study of new NF-κB-inducing kinase (NIK) ligands derived from the imidazolone scaffold

Francisco Maqueda,^1^ Lara Valiño-Rivas,^2^ Ana Milián,^1^ Sara Gutiérrez-Hernández,^1^ José Luis Aceña,^1,3,^* Javier Garcia-Marin,^1,3,^* Mª Dolores Sánchez-Niño,^2,3,4,^* Juan J. Vaquero,^1,3^ Alberto Ortiz,^2,3,^*

1 Departamento de Química Orgánica y Química Inorgánica, Universidad de Alcalá (IRYCIS), Instituto de Investigación Química “Andrés M. Del Río” (IQAR), 28805-Alcalá de Henares, Madrid, Spain.

2 Departamento de Nefrología e Hipertensión, IIS-Fundación Jiménez Díaz UAM, Madrid, Spain.

3 RICORS2040, Madrid, Spain

4 Departamento de Farmacología, Facultad de Medicina, Universidad Autónoma de Madrid, 28049 Madrid, Spain

*Correspondence:

Javier Garcia-Marin, Department of Organic and Inorganic Chemistry, University of Alcalá (IRYCIS), Spain

Email: [javier.garciamarin@uah.es](mailto:javier.garciamarin@uah.es)

Alberto Ortiz, Department of Nephrology and Hypertension, IIS-Fundacion Jimenez Diaz UAM, 28040 Madrid, Spain

Email:  [aortiz@fjd.es](mailto:javier.garciamarin@uah.es)

**Table 1.** Human recombinant NIK affinity of synthesized compounds.

| **Cpd. No.** | **InChI** | **Kd (µM)** |
| --- | --- | --- |
| 3a | InChI=1S/C25H22N6O2/c1-33-12-11-31-23(16-7-3-2-4-8-16)30-21(24(31)32)13-20-22(17-14-27-25(26)28-15-17)18-9-5-6-10-19(18)29-20/h2-10,13-15,29H,11-12H2,1H3,(H2,26,27,28)/b21-13- | >10 |
| 3b | InChI=1S/C19H18N6O2/c1-27-7-6-25-11-23-16(18(25)26)8-15-17(12-9-21-19(20)22-10-12)13-4-2-3-5-14(13)24-15/h2-5,8-11,24H,6-7H2,1H3,(H2,20,21,22)/b16-8- | 1.5 |
| 3c | InChI=1S/C20H20N6O2/c1-12-24-17(19(27)26(12)7-8-28-2)9-16-18(13-10-22-20(21)23-11-13)14-5-3-4-6-15(14)25-16/h3-6,9-11,25H,7-8H2,1-2H3,(H2,21,22,23)/b17-9- | 5.1 |
| 3d | InChI=1S/C24H21N7O2/c1-33-11-10-31-22(15-6-8-26-9-7-15)30-20(23(31)32)12-19-21(16-13-27-24(25)28-14-16)17-4-2-3-5-18(17)29-19/h2-9,12-14,29H,10-11H2,1H3,(H2,25,27,28)/b20-12- | 0.98 |
| 3e | InChI=1S/C23H20N6O3/c1-31-10-8-29-21(19-7-4-9-32-19)28-18(22(29)30)11-17-20(14-12-25-23(24)26-13-14)15-5-2-3-6-16(15)27-17/h2-7,9,11-13,27H,8,10H2,1H3,(H2,24,25,26)/b18-11- | 2.8 |
| 3f | InChI=1S/C23H20N6O2S/c1-31-9-8-29-21(19-7-4-10-32-19)28-18(22(29)30)11-17-20(14-12-25-23(24)26-13-14)15-5-2-3-6-16(15)27-17/h2-7,10-13,27H,8-9H2,1H3,(H2,24,25,26)/b18-11- | 6.1 |
| 3g | InChI=1S/C23H20N6O3/c1-31-9-7-29-21(14-6-8-32-13-14)28-19(22(29)30)10-18-20(15-11-25-23(24)26-12-15)16-4-2-3-5-17(16)27-18/h2-6,8,10-13,27H,7,9H2,1H3,(H2,24,25,26)/b19-10- | 4.5 |
| 3h | InChI=1S/C23H20N6O2S/c1-31-8-7-29-21(14-6-9-32-13-14)28-19(22(29)30)10-18-20(15-11-25-23(24)26-12-15)16-4-2-3-5-17(16)27-18/h2-6,9-13,27H,7-8H2,1H3,(H2,24,25,26)/b19-10- | 5.5 |
| 3i | InChI=1S/C25H21ClN6O2/c1-34-10-9-32-23(15-5-4-6-17(26)11-15)31-21(24(32)33)12-20-22(16-13-28-25(27)29-14-16)18-7-2-3-8-19(18)30-20/h2-8,11-14,30H,9-10H2,1H3,(H2,27,28,29)/b21-12- | >10 |
| 3j | InChI=1S/C25H21BrN6O2/c1-34-11-10-32-23(15-6-8-17(26)9-7-15)31-21(24(32)33)12-20-22(16-13-28-25(27)29-14-16)18-4-2-3-5-19(18)30-20/h2-9,12-14,30H,10-11H2,1H3,(H2,27,28,29)/b21-12- | >10 |
| 3k | InChI=1S/C26H21F3N6O2/c1-37-11-10-35-23(15-6-8-17(9-7-15)26(27,28)29)34-21(24(35)36)12-20-22(16-13-31-25(30)32-14-16)18-4-2-3-5-19(18)33-20/h2-9,12-14,33H,10-11H2,1H3,(H2,30,31,32)/b21-12- | >10 |
| 3l | InChI=1S/C27H27N7O2/c1-33(2)19-10-8-17(9-11-19)25-32-23(26(35)34(25)12-13-36-3)14-22-24(18-15-29-27(28)30-16-18)20-6-4-5-7-21(20)31-22/h4-11,14-16,31H,12-13H2,1-3H3,(H2,28,29,30)/b23-14- | >10 |
| 3m | InChI=1S/C26H24N6O3/c1-34-11-10-32-24(16-6-5-7-18(12-16)35-2)31-22(25(32)33)13-21-23(17-14-28-26(27)29-15-17)19-8-3-4-9-20(19)30-21/h3-9,12-15,30H,10-11H2,1-2H3,(H2,27,28,29)/b22-13- | >10 |
| 3n | InChI=1S/C26H24N6O3/c1-34-12-11-32-24(16-7-9-18(35-2)10-8-16)31-22(25(32)33)13-21-23(17-14-28-26(27)29-15-17)19-5-3-4-6-20(19)30-21/h3-10,13-15,30H,11-12H2,1-2H3,(H2,27,28,29)/b22-13- | >10 |

**Table 2.** Cell viability after 24h in human fibroblast (HFF1 cell line, from American Type Culture Collection) at 10 µM.

| **Cpd. No.** | **InChI** | **% Viability** | **SD** |
| --- | --- | --- | --- |
| 3b | InChI=1S/C19H18N6O2/c1-27-7-6-25-11-23-16(18(25)26)8-15-17(12-9-21-19(20)22-10-12)13-4-2-3-5-14(13)24-15/h2-5,8-11,24H,6-7H2,1H3,(H2,20,21,22)/b16-8- | 84.41 | 0.06 |
| 3c | InChI=1S/C20H20N6O2/c1-12-24-17(19(27)26(12)7-8-28-2)9-16-18(13-10-22-20(21)23-11-13)14-5-3-4-6-15(14)25-16/h3-6,9-11,25H,7-8H2,1-2H3,(H2,21,22,23)/b17-9- | 112.6 | 0.22 |
| 3d | InChI=1S/C24H21N7O2/c1-33-11-10-31-22(15-6-8-26-9-7-15)30-20(23(31)32)12-19-21(16-13-27-24(25)28-14-16)17-4-2-3-5-18(17)29-19/h2-9,12-14,29H,10-11H2,1H3,(H2,25,27,28)/b20-12- | 101.84 | 0.39 |
| 3e | InChI=1S/C23H20N6O3/c1-31-10-8-29-21(19-7-4-9-32-19)28-18(22(29)30)11-17-20(14-12-25-23(24)26-13-14)15-5-2-3-6-16(15)27-17/h2-7,9,11-13,27H,8,10H2,1H3,(H2,24,25,26)/b18-11- | 34.78 | 0.09 |
| 3f | InChI=1S/C23H20N6O2S/c1-31-9-8-29-21(19-7-4-10-32-19)28-18(22(29)30)11-17-20(14-12-25-23(24)26-13-14)15-5-2-3-6-16(15)27-17/h2-7,10-13,27H,8-9H2,1H3,(H2,24,25,26)/b18-11- | 109.38 | 0.09 |
| 3g | InChI=1S/C23H20N6O3/c1-31-9-7-29-21(14-6-8-32-13-14)28-19(22(29)30)10-18-20(15-11-25-23(24)26-12-15)16-4-2-3-5-17(16)27-18/h2-6,8,10-13,27H,7,9H2,1H3,(H2,24,25,26)/b19-10- | 84.51 | 0.06 |
| 3h | InChI=1S/C23H20N6O2S/c1-31-8-7-29-21(14-6-9-32-13-14)28-19(22(29)30)10-18-20(15-11-25-23(24)26-12-15)16-4-2-3-5-17(16)27-18/h2-6,9-13,27H,7-8H2,1H3,(H2,24,25,26)/b19-10- | 111.49 | 0.02 |
