# Supplementary material for: Identification and study of new NF‐κB‐inducing kinase ligands derived from the imidazolone scaffold
Source: Arch Pharm (Weinheim). 2024 Nov 27;358(1):e2400614. doi: 10.1002/ardp.202400614 (PMC11704032; doi:10.1002/ardp.202400614)
Supplement: Supplementary file 2 — Supporting information. [file ARDP-358-e2400614-s001.docx]

**Identification and study of new NF-κB-inducing kinase (NIK) ligands derived from the imidazolone scaffold**

Francisco Maqueda-Zalaya,^1^ Lara Valiño-Rivas,^2^ Ana Milián,^1^ Sara Gutiérrez,^1^ Jose Luis Aceña,^1,3,^* Javier Garcia-Marin,^1^* Mª Dolores Sánchez-Niño,^2,3,4,^* Juan J. Vaquero,^1,3^ Alberto Ortiz,^2,3,^*

1 Departamento de Química Orgánica y Química Inorgánica, Universidad de Alcalá (IRYCIS), Instituto de Investigación Química “Andrés M. Del Río” (IQAR), 28805-Alcalá de Henares, Madrid, Spain.

2 Departamento de Nefrología e Hipertensión, IIS-Fundación Jiménez Díaz UAM, Madrid, Spain.

3 RICORS2040, Madrid, Spain

4 Departamento de Farmacología, Facultad de Medicina, Universidad Autónoma de Madrid, 28049 Madrid, Spain

*Correspondence:

Javier Garcia-Marin, Department of Organic and Inorganic Chemistry, University of Alcalá (IRYCIS), Spain

Email: [javier.garciamarin@uah.es](mailto:javier.garciamarin@uah.es)

Alberto Ortiz, Department of Nephrology and Hypertension, IIS-Fundacion Jimenez Diaz UAM, 28040 Madrid, Spain

Email:  [aortiz@fjd.es](mailto:javier.garciamarin@uah.es)

| **Table of Contents** | |
| --- | --- |
| **Affinity curves (Fig S1)** | **S1** |
| **Computational details (Fig S2, S3 & S4)** | **S2** |
| **^1^H-NMR & ^13^C-RMN spectra of final products** | **S4** |
| **HPLC of representative compounds** | **S37** |


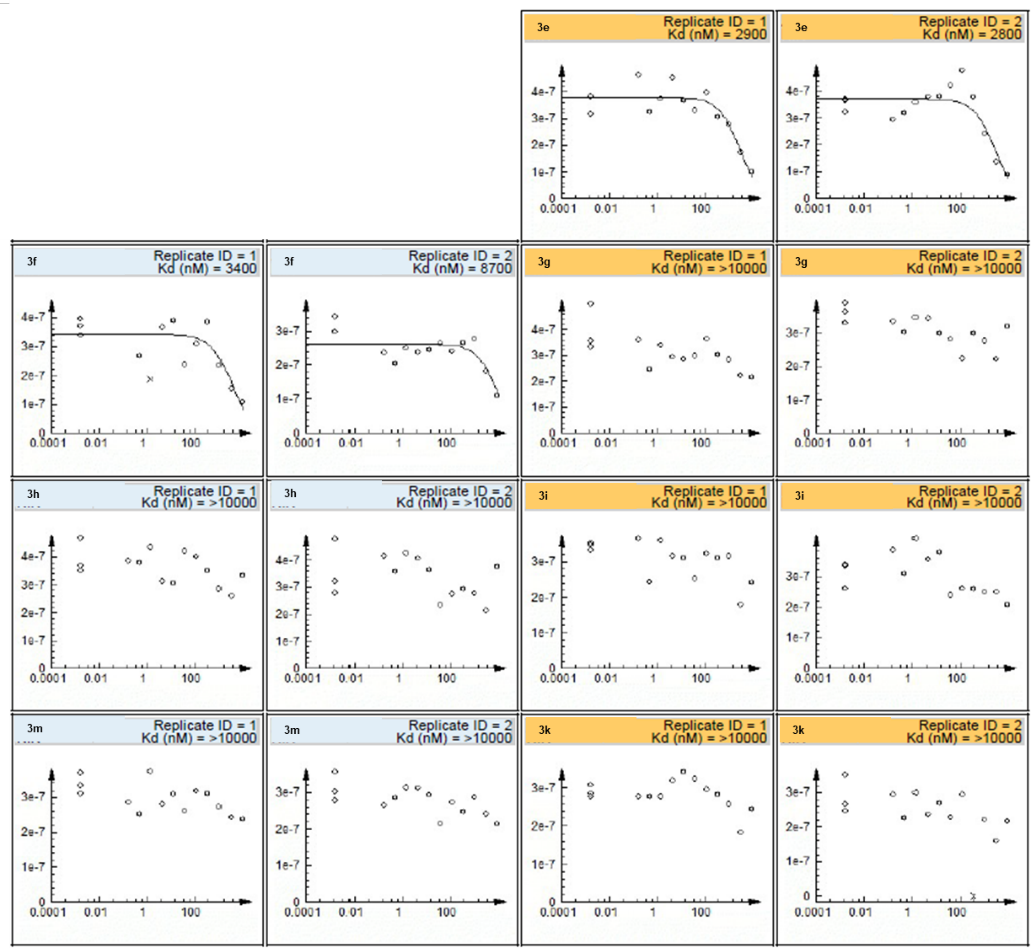


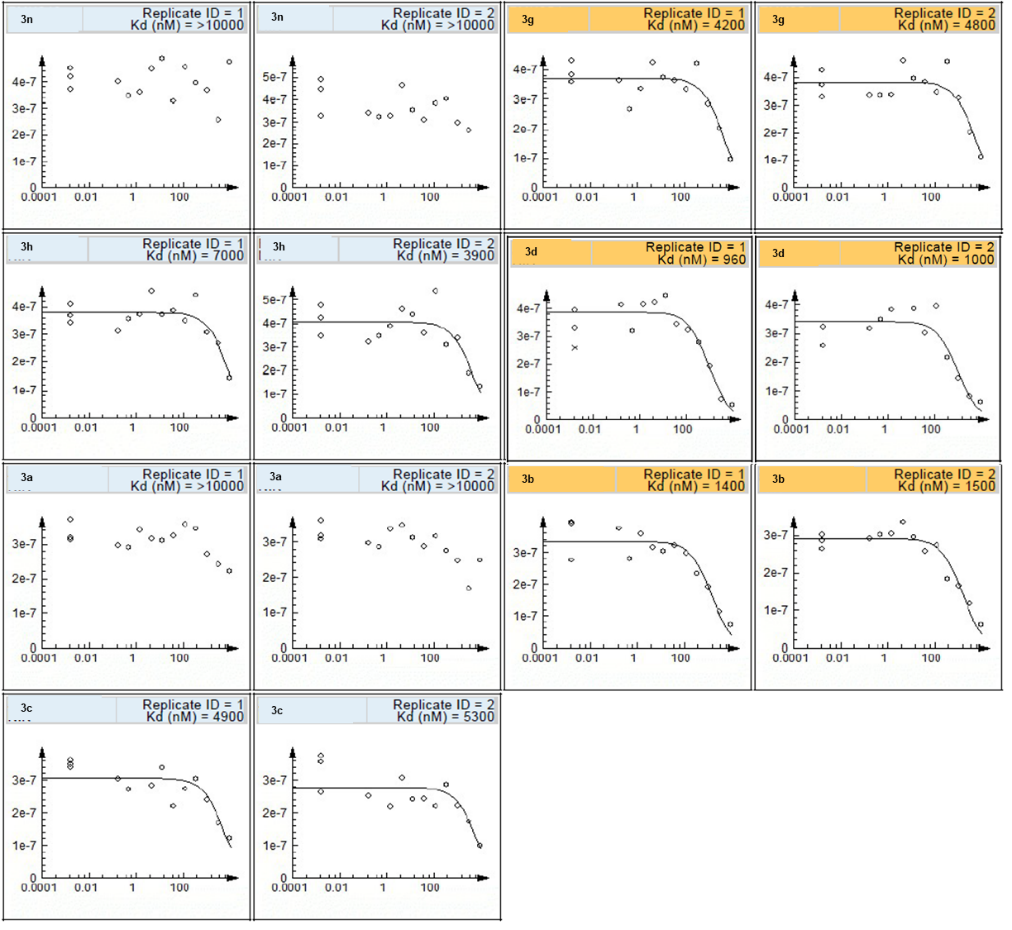


**Figure S 1.** Affinity measurement plots. The amount of kinase measured by qPCR (Signal; y-axis) is plotted against the corresponding compound concentration in nM in log10 scale (x-axis). Data points marked with an "x" were not used for Kd determination.


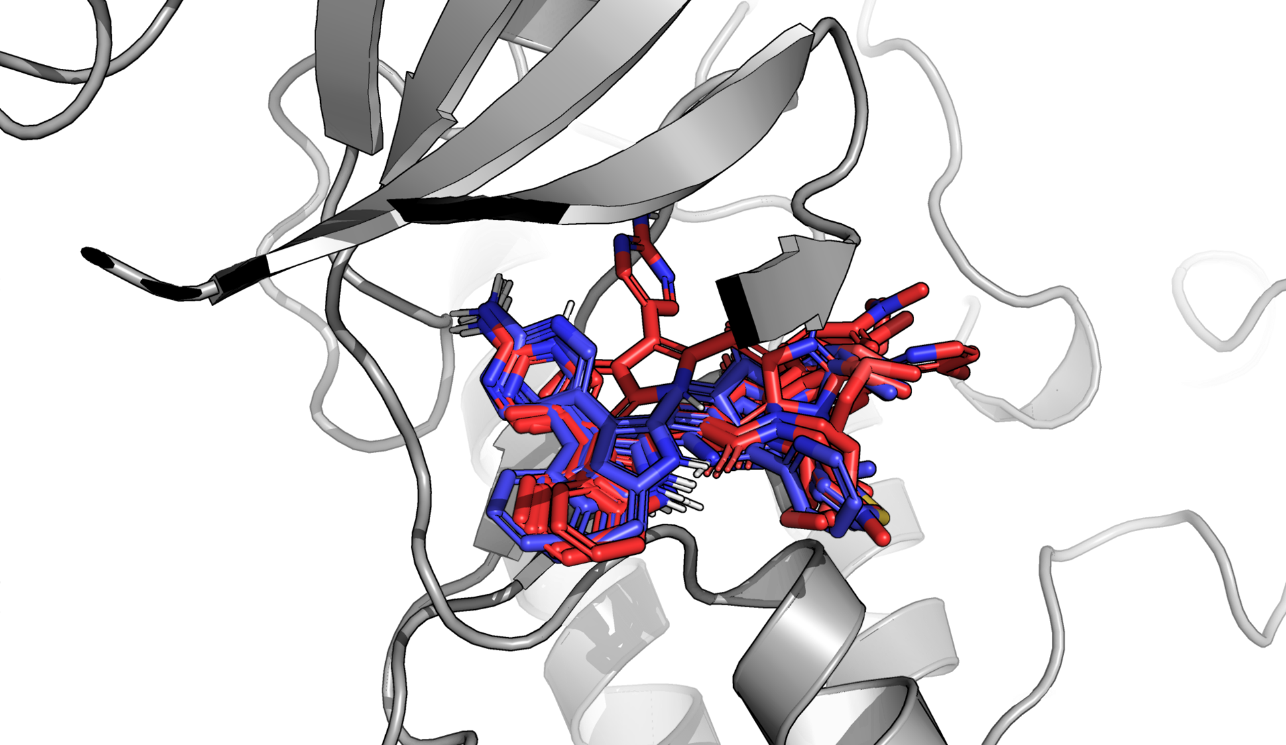


**Figure S 2.** Superposition of docking poses for all azolo-imidazolidenone derivatives. In blue those compounds wich showed affinity against NIK and in red, those which did not bind to the protein.


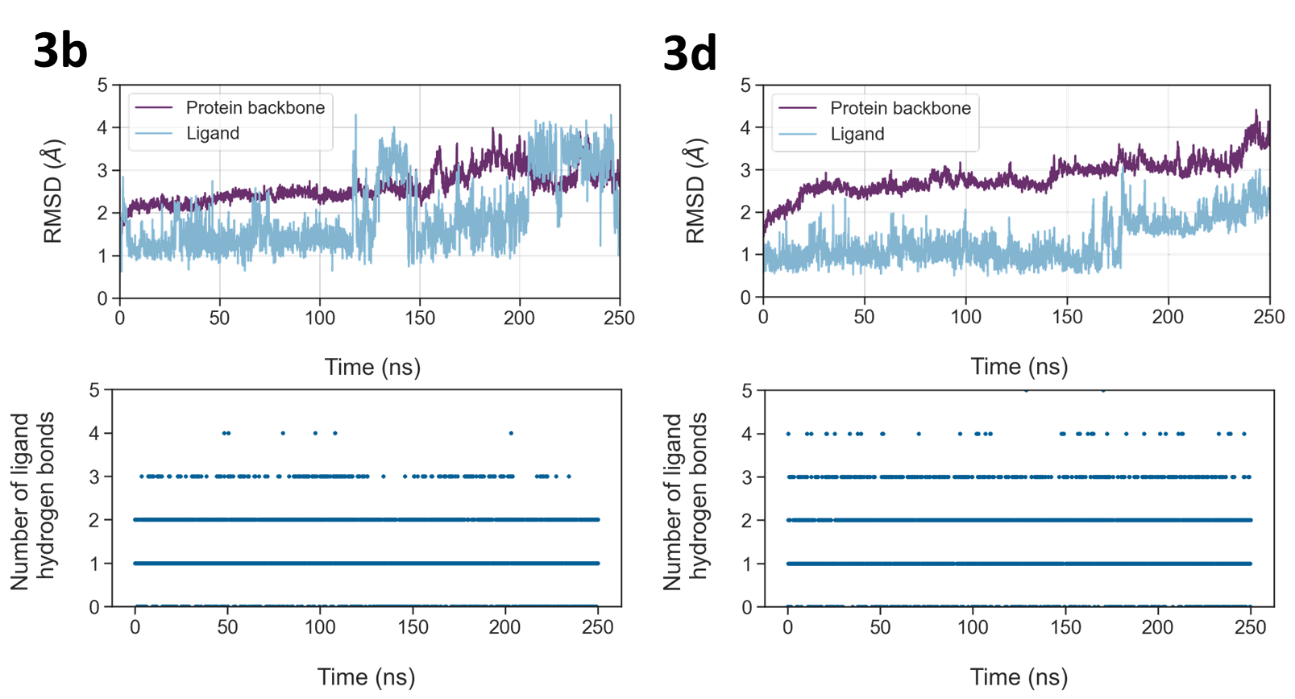


**Figure S 3.** (Up) Protein backbone and ligand heavy atoms RMSD across the MD trajectory and (down) number of hydrogen bonds stablished between ligand for **3b** (left) and **3d** (right) with the protein.


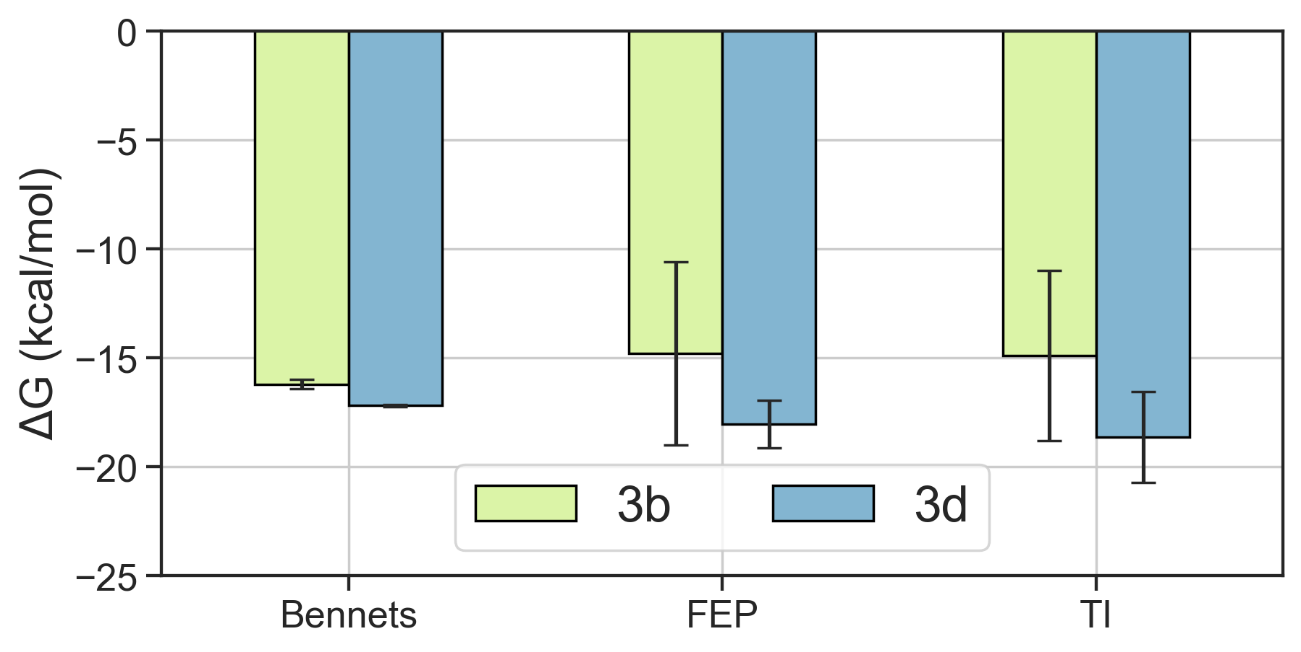


**Figure S4.** Binding free energy calculated from WaterSwap simulations using Bennet’s Acceptance ratio (Bennets), Free Energy perturbation (FEP) and Thermodinamic Integration (TI).

**^1^H-NMR (300 MHz, CDCl_3_)**

**^13^C-NMR (75 MHz, CDCl_3_)**

**^1^H-NMR (300 MHz, CDCl_3_)**

**^13^C-NMR (75 MHz, CDCl_3_)**

**^1^H-NMR (300 MHz, CDCl_3_)**

**^13^C-NMR (75 MHz, CDCl_3_)**

**^1^H-NMR (300 MHz, CDCl_3_)**

**^13^C-NMR (75 MHz, CDCl_3_)**

**^1^H-NMR (300 MHz, CDCl_3_)**

**^13^C-NMR (75 MHz, CDCl_3_)**

**^1^H-NMR (300 MHz, CDCl_3_)**

**^13^C-NMR (75 MHz, CDCl_3_)**

**^1^H-NMR (300 MHz, CDCl_3_)**

**^13^C-NMR (75 MHz, CDCl_3_)**

**^1^H-NMR (300 MHz, CDCl_3_)**

**^13^C-NMR (75 MHz, CDCl_3_)**

**^1^H-NMR (300 MHz, CDCl_3_)**

**^13^C-NMR (75 MHz, CDCl_3_)**

**^1^H-NMR (300 MHz, CDCl_3_)**

**^13^C-NMR (75 MHz, CDCl_3_)**

**^19^F-NMR (282 MHz, CDCl_3_)**

**^1^H-NMR (300 MHz, CDCl_3_)**

**^13^C-NMR (75 MHz, CDCl_3_)**

**^1^H-NMR (300 MHz, CDCl_3_)**

**^13^C-NMR (400 MHz, CDCl_3_)**

**^1^H-NMR (300 MHz, CDCl_3_)**

**^13^C-NMR (75 MHz, CDCl_3_)**

**^1^H-NMR (300 MHz, CDCl_3_)**

**^13^C-NMR (75 MHz, CDCl_3_)**

**^1^H-NMR (300 MHz, CDCl_3_)**

**^13^C-NMR (75 MHz, CDCl_3_)**

**^1^H-NMR (400 MHz, DMSO)**

**^13^C-NMR (101 MHz, DMSO)**

**HPLC OF MOST IMPORTANT COMPOUNDS**

**
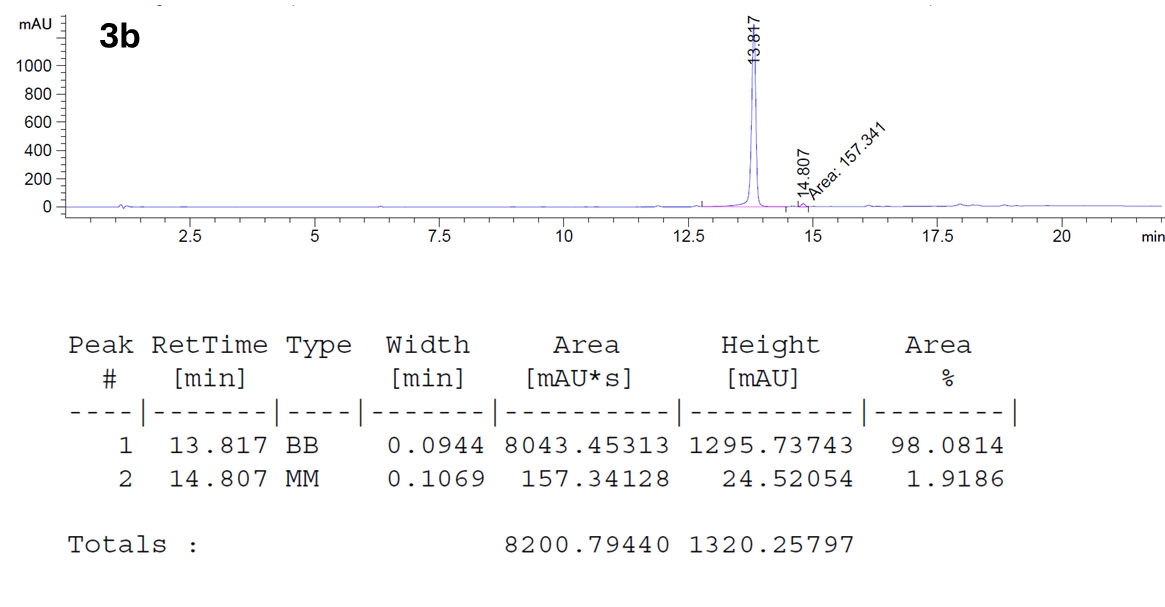
**

**
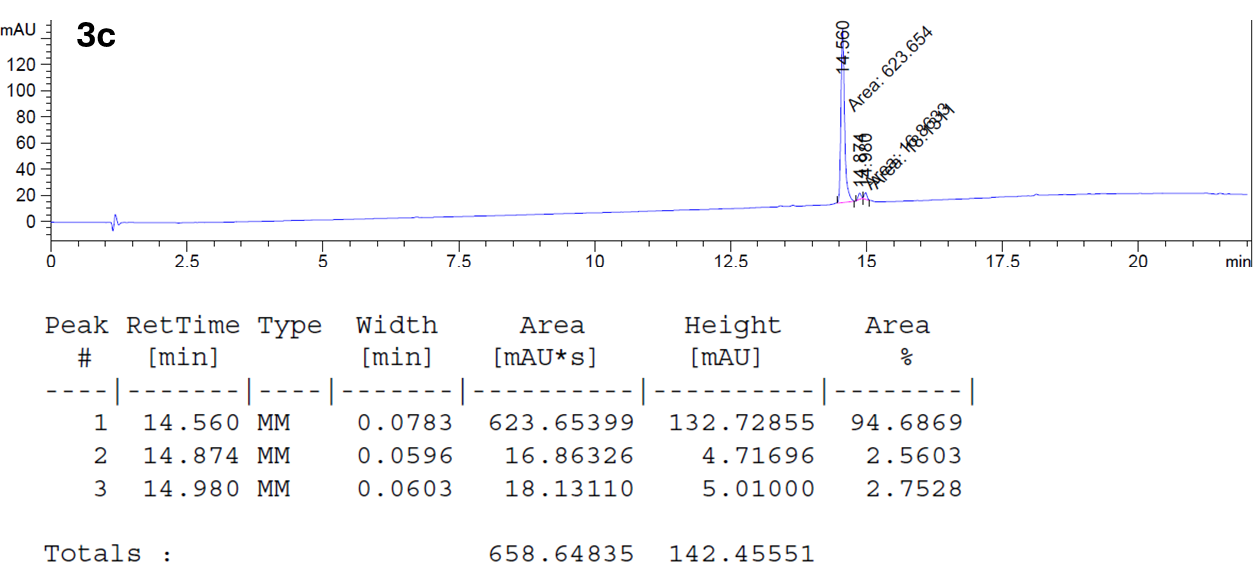
**

**
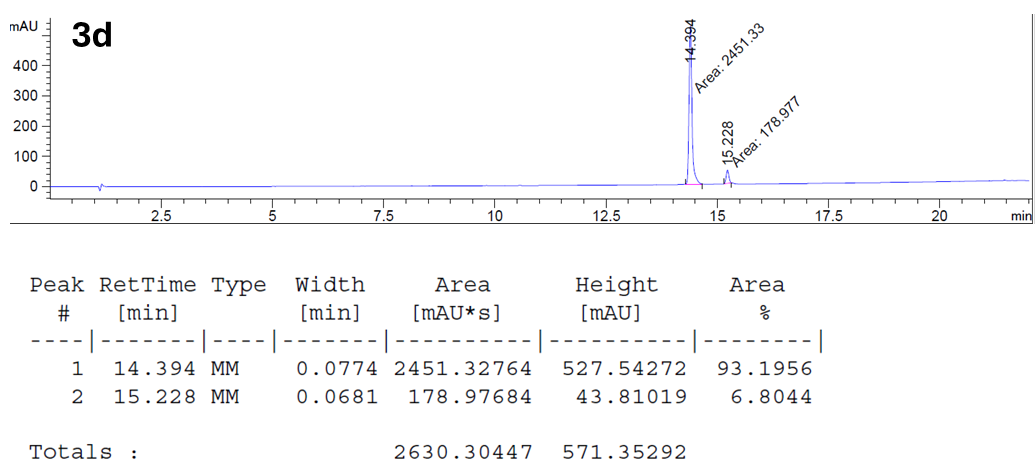
**
